# Inosine induces context-dependent recoding and translational stalling

**DOI:** 10.1093/nar/gky1163

**Published:** 2018-11-20

**Authors:** Konstantin Licht, Markus Hartl, Fabian Amman, Dorothea Anrather, Michael P Janisiw, Michael F Jantsch

**Affiliations:** 1 Center for Anatomy and Cell Biology, Medical University of Vienna, Schwarzspanierstrasse 17, A-1090 Vienna, Austria; 2 Mass Spectrometry Facility, Max F. Perutz Laboratories (MFPL), Vienna Biocenter (VBC), Dr. Bohr-Gasse 3, A-1030 Vienna, Austria; 3 Institute of Theoretical Biochemistry, University of Vienna, Währingerstrasse 17, A-1090 Vienna, Austria

## Abstract

RNA modifications are present in all classes of RNAs. They control the fate of mRNAs by affecting their processing, translation, or stability. Inosine is a particularly widespread modification in metazoan mRNA arising from deamination of adenosine catalyzed by the RNA-targeting adenosine deaminases ADAR1 or ADAR2. Inosine is commonly thought to be interpreted as guanosine by cellular machines and during translation. Here, we systematically test ribosomal decoding using mass spectrometry. We show that while inosine is primarily interpreted as guanosine it can also be decoded as adenosine, and rarely even as uracil. Decoding of inosine as adenosine and uracil is context-dependent. In addition, mass spectrometry analysis indicates that inosine causes ribosome stalling especially when multiple inosines are present in the codon. Indeed, ribosome profiling data from human tissues confirm inosine-dependent ribosome stalling *in vivo*. To our knowledge this is the first study where decoding of inosine is tested in a comprehensive and unbiased way. Thus, our study shows novel, unanticipated functions for inosines in mRNAs, further expanding coding potential and affecting translational efficiency.

## INTRODUCTION

The four nucleotides of RNA can be chemically modified in multiple ways. Today, >150 different types of modifications are known (1). RNA-modifications can affect the fate and function of all classes of RNAs (2). Moreover, modifications can affect RNA-processing, stability, localization, and translation (2–4). Several modifications can be ‘written’, ‘read’ and ‘erased’. Therefore, by analogy to the reversible modifications found in DNA, RNA modifications have recently been termed the ‘epitranscriptome’ (5).

Adenosine to inosine RNA editing (A-to-I editing) is among the most prevalent epitranscriptomic changes found in metazoa. Here, adenosines are deaminated to inosine by adenosine deaminases acting on RNA (ADARs) (6–8). The deamination reaction changes the base-pairing potential of the nucleotide. Removal of the hydrogen-donating amino group at the C6 position of adenine, leaves a hydrogen accepting oxygen. The conversion of adenosine can have manifold consequences, ranging from re-coding of transcripts, over changes in miRNA-targeting, the modulation of alternative splicing, to effects on innate immunity (2,6,7). Millions of A-to-I editing sites are present in the human transcriptome (9–14). Most of the sites locate to non-coding regions of transcripts like introns or UTRs. However, over 1000 sites are found in coding regions (CDS) of transcripts. For instance, a recent mining of RNA-seq data from different human tissues found 1741 A-to-I editing events in CDS regions (14). A-to-I editing events in CDS can recode mRNAs and lead to the incorporation of amino acids into proteins that are not encoded at the DNA level. Consequently, mRNA recoding by A-to-I editing can have dramatic consequences for health and disease (15–19). A-to-I editing in protein-coding targets is very abundant in the central nervous system (20). Consequently, aberrant A-to-I editing is frequently linked to different neurological disorders (20).

Two catalytically active A-to-I editing enzymes, ADAR1 and ADAR2 have been identified in mammals. ADAR1 deficiency affects the innate immune response seemingly by marking endogenous structured RNAs as ‘self’ (21,22). ADAR2 editing on the other hand targets mostly different protein-coding transcripts (23). A very important target of ADAR2 is the mRNA encoding glutamate receptor subunit 2 (*Gria2*). ADAR2 null mice are lethal but can be rescued by expression of a constitutively edited version of *Gria2* (24). Still, ADAR2 has been shown to recode many other mRNAs (20).

ADAR3, the third member of the ADAR family is enzymatically inactive (25,26). Still, an ADAR3 knockout mouse lacking both dsRNA-binding domains (dsRBDs) shows a modest cognitive phenotype impacting memory and learning, possibly by competing with the editing-active ADARs for access to editing sites or by binding to double stranded RNAs (27,28).

**Figure 1.**
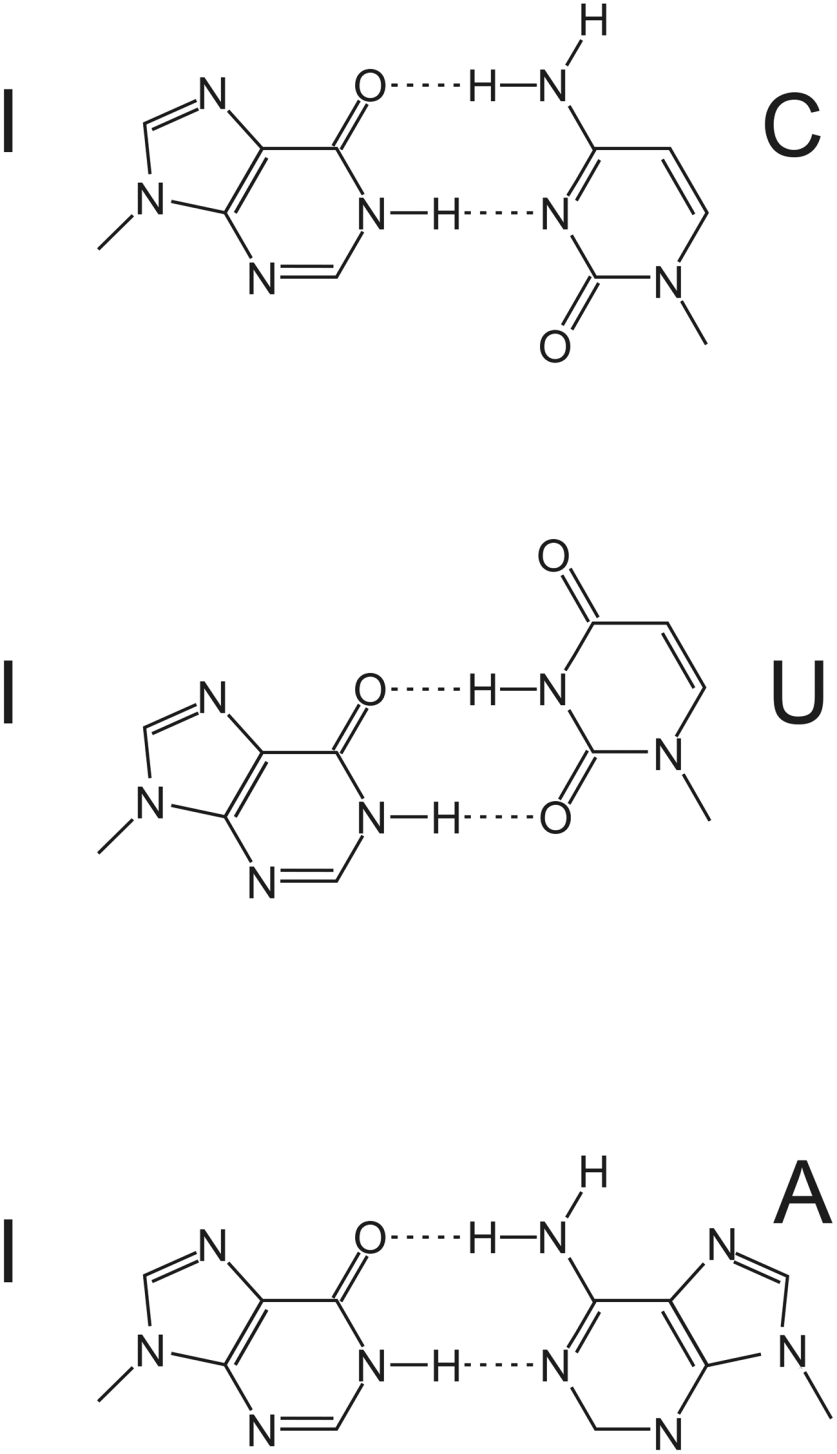
Inosine can basepair with cytidine, uracil or adenine, in each case forming two hydrogen bonds.

Inosine is commonly thought to be interpreted as guanosine by cellular machines (29). However, the base-pairing potential of guanosine and inosine differ due to the lacking amino group at the C6 position of inosine. Therefore, while guanosine and inosine can base-pair with C and U, inosine may additionally base-pair with A (Figure 1). Still, I:C base-pairs are most stable followed by I:A and I:U base-pairs (30).

The broad base-pairing potential of inosine can be best observed at position 34 of 8 cytosolic tRNAs where an inosine is located at the 5′ position (wobble position) of the anticodon (31,32). At the wobble position inosine can pair with C, A or U as originally proposed by Crick (33). Interestingly, non-Watson/Crick base-pairing is also tolerated during codon/anti-codon base-pairing outside the wobble position – at least transiently (34). Moreover, inosine does not destabilize short DNA-helices when it pairs with either C, U or A (35,36). Finally, guanosine or inosine in miRNAs exhibited significant differences in mRNA-silencing efficiencies (37). Taken together, this suggests that inosine does not simply mimic guanosine during translation and argues for a more complex translational decoding.

Here, we test the decoding of inosine in the context of different codons using *in vitro* transcribed reporter RNAs followed by *in vitro* translation and mass spectrometry. We demonstrate that inosine is primarily decoded as guanosine but also as adenosine or uracil. In addition, we find unexpected translational stalling when inosine is present in the codon. This is further supported by analysis of different ribosome-sequencing datasets indicating that inosine influences translation kinetics *in vivo*.

## MATERIALS AND METHODS

### Cloning of test-constructs

A pUC18 vector was linearized with KpnI and EcoRI and a 40 bp long poly(A) tail plus a downstream BglII site was introduced using two annealed oligonucleotides with overhangs matching the KpnI and EcoRI sites (38). Subsequently, a gBlocks^®^ Gene Fragment (Integrated DNA Technologies, Coralville, IA, USA) coding for a T7-promoter, 5′UTR, Flag-TAG, XhoI restriction site, NheI restriction site, spacer encoding additional amino acids, and a 3′UTR was added. Test-sequences were introduced via XhoI and NheI sites using annealed oligonucleotides with complementary overhangs. Finally, all reporter plasmids harbored the sequence: 5′-TAATACGACTCACTATAGGGTCATACAACATACAAACATACTACACATACAAACACACAATACAACAACATACATACAACAATCTTAATTAACACCACC**ATG**GACTATAAAGACGATGACGATAAACTCTCGAAATCATCATTCTACTCCTTAACATCC**NNN**TCTAACATATCCAAACTAGCCGAATTCATCATAATTTTAAACTACACATTCATCTTATTATTAAACATCTCCACCTATCTATTACTTTCCTTATCATCCTCTTACCCATGCCAC**TAA**TGATAAGAATTCTAATAACACTATACTATTTCTTACTATCCGGGTACTGCGCAAAAAAAAAAAAAAAAAAAAAAAAAAAAAAAAAAAAAAAAAGATCT-3′ The start-codon, the test-codon (NNN), and the stop-codon are shown in bold letters. The sequence coding for the FLAG tag is underlined. In case of the AGA-test codon, the BglII site used for linearization of the plasmids prior to *in vitro* transcription was replaced by an EcoRV-site using site-directed mutagenesis.

### In vitro transcription and vitro translation

Reporter plasmids were linearized using BglII or EcoRV in case of the plasmid coding for the AGA-codon. *In vitro* transcription was done with NEB HiScribe™ T7 High Yield RNA Synthesis Kit (New England Biolabs, Ipswich, MA) according to the manufacturer's instructions for capped RNA synthesis with the following modifications: synthesis was carried out for 16 h overnight in a 37°C incubator to prevent evaporation using 10 mM of ITP instead of GTP and 2 mM of m^7^G cap analog (NEB #S1404S) followed by DNaseI digestion (Thermo Fisher Scientific, MA, Waltham, #EN0521) using 5 units. 400 ng of transcript was used for *in vitro* translation using rabbit reticulocyte lysate (RRL) treated with micrococcal nuclease (Promega, Fitchburg, WI #L4960). Reactions were carried out in a 100 μl total volume containing 70 μl RRL, 10 mM creatine phosphate, 50 μg/ml creatine phosphokinase, 50 μg/ml calf liver tRNA, 79 mM potassium acetate, 0.5 mM magnesium acetate, 0.02 mM hemin, and a complete mix of amino acids (minus Met mixed with minus Leu) at a concentration of 1 mM. Following incubation at 30°C for 1.5 h, the lysate was supplemented with 300 μl TBS (50 mM Tris/Cl pH 7.4, 150 mM NaCl) and incubated for 60 min at room temperature with 30 μl of Anti-FLAG^®^ M2 Magnetic Beads (Sigma-Aldrich, St. Louis, MO) on a rotating wheel. Beads were washed 3 times with 900 μl TBS-NDSE (50 mM Tris/Cl pH 7.4, 150 mM NaCl, 1% NP-40, 1% deoxycholate, 0.1% SDS, 2 mM EDTA) and 5 times with TBS.

### On-bead proteolytic digest

Peptide bound magnetic beads were resuspended in 50 mM ammonium bicarbonate. In case of cysteine containing sequences proteins were reduced with DTT (10 mM) and free thiols were alkylated with 50 mM iodo acetamide in the dark for 30 min, followed by digestion with 100 ng Lysyl Endopeptidase (LysC, Wako Chemicals) for 3 h at room temperature. Digestion was stopped by adding trifluoroacetic acid. The peptide solutions were desalted on custom-made C18 stagetips (39).

### Liquid chromatography mass spectrometry and data analysis

Peptides were separated on an Ultimate 3000 RSLC nano-flow chromatography system (Thermo Fisher Scientific), using a pre-column for sample loading (PepMapAcclaim C18, 2 cm × 0.1 mm, 5 μm, Dionex-Thermo-Fisher) and a C18 analytical column (PepMapAcclaim C18,  50  cm ×  0.75  mm, 2 μm, Dionex-Thermo-Fisher), applying a linear gradient from 2 to 35% solvent B (80% acetonitrile, 0.1% formic acid; solvent A 0.1% formic acid) at a flow rate of 230 nl min^−1^ over 60 min. Eluting peptides were analysed on a Q Exactive HF (or HFX) Orbitrap mass spectrometer, equipped with a Proxeon nanospray source (all Thermo Fisher Scientific), operated in a data-dependent mode. Survey scans were obtained in a mass range of 380–1650 *m/z* with lock mass on, at a resolution of 120 000 at 200 *m/z* and an AGC target value of 3E6. The 10 most intense ions were selected with an isolation width of 2 Da, fragmented in the HCD cell at 27% collision energy and the spectra recorded at a target value of 1E5 and a resolution of 30000. Peptides with a charge of +1 were excluded from fragmentation, the peptide match and exclude isotope features were enabled and selected precursors were dynamically excluded from repeated sampling for 15 s. Raw data were processed using the MaxQuant software package (40) (http://www.maxquant.org/) and searched against the target sequences in the rabbit uniprot background (www.uniprot.org). The search was performed with full LysC specificity and a maximum of two missed cleavages. Carbamidomethylation of cysteine was set as fixed, oxidation of methionine and N-terminal protein acetylation as variable modifications—all other parameters were set to default. All target peptide identifications were inspected manually. Ion intensity chromatograms of the peptides were extracted and quantified with Skyline-daily 4.1.1.18179 (41). Precursor ion traces for quantification were accepted only if they matched the retention time window of the corresponding MS/MS scans used for identification, were derived from a mono-isotopic precursor with the correct charge state and a mass accuracy <5 ppm, and displayed an isotope dot-product >0.95.

### Cloning and expression of the concatemers used for normalization

To normalize intensities observed for individual peptides by mass spectrometry all peptide permutations were expressed as an N-terminally GST and C-terminally 6xHis-tagged concatenated fusion protein in *Escherichia coli*. For cloning two gBlocks^®^ Gene Fragments (Integrated DNA Technologies, Coralville, IA) coding for all peptides in two different orders were cloned into the pGEX-1 (GE Healthcare, #27-1542-01) plasmid using BamHI and EcoRI sites. For protein expression in *E. coli* BL21(DE3), cultures were grown to OD(600) = 0.6 and induced by addition of 1 mM IPTG final concentration. Cultures were incubated for 16 h at 20°C and harvested subsequently. Whole cell extracts were prepared by boiling *E. coli* pellets directly in Laemmli SDS sample loading buffer. Expression of fusion proteins was verified by western blotting using Goat anti-GST (Rockland, #600-101-200, 1:5000) and Rabbit anti-His antibodies (Cell Signaling Technologies, #12698). For normalization cell extracts were separated on 15% SDS PAGE, stained with 0.25% Coomassie R250. Protein bands were excised and subjected to mass spectrometry analysis. In-gel digest was done using Lysyl Endopeptidase as previously described (42).

### Amplicon sequencing and analysis of NGS data

100 ng of *in vitro* transcribed RNA samples were subjected to DNaseI digestion (2 U, 30 min, 37°C, Thermo Scientific, #EN0521). The enzyme was inactivated for 10 minutes at 65°C. 5 μl RNA were primed with random hexamers (Integrated DNA Technologies, Leuven, Belgium) and reverse transcribed with Maxima RT (Thermo Scientific, #EP0742) according to the manufacturer's recommendations. RT reactions were amplified in 25 cycles of High Fidelity PCR (Phusion HSII, Thermo Scientific, #F549) with target specific primers. PCR amplicons were purified using Wizard PCR Clean-Up (Promega, #A9282). Eluates were diluted 10-fold and subjected to 25 cycles of Adaptor-PCR-2 to introduce Illumina barcodes. 20% of each PCR reaction was checked on 3% and 1.5% TAE agarose gel. 4 μl aliquots of each Adaptor-PCR-2 reaction were pooled and subjected to size selection purification using Agencourt AMPure XP Beads (Beckman Coulter, #A63881) following library preparation kit recommendations (NEBNext UltraTM Directional RNA Library Prep Kit for Illumina, NEB, #E7420). Final library concentration was determined by fluorometric measurement (Qubit3.0 dsDNA HS Assay Kit, Invitrogen, #Q32854). Following sequencing on an Illumina HiSeq2500 machine, reads were mapped against the amplicon reference sequences using BWA-MEM with reduced clipping penalty of 1 (43) (https://arxiv.org/abs/1303.3997). RNA–DNA differences between the reference and the read sequence were quantified by bam-readcount (https://github.com/genome/bam-readcount).

### Analysis of the ribosome profiling data

Adapter sequences clipping and quality trimming was performed with cutadapt (44). Reads were mapped against human reference genome (hg38) using short read mapper STAR, multimappers were removed and the replicas pooled together (45). Editing levels of all exonic editing sites annotated in the database REDIportal (46) were calculated using bam-readcount from the mRNA-seq data. Genomic read coverage plots from the ribosome profiling data were produced using bedtools genomecov (47,48). Editing sites were intersected with human coding sequence annotation (GENCODE basic v22). If several CDS matched, the longest transcript was chosen. Transcripts with two or more editing sites in close vicinity (±5 nt) were not considered. For the remaining transcripts the transcript coverage was assembled from the genomic coverage data. The total ribosome profiling signal for each editing site was normalized by the total signal in the ±500 nt range surrounding of the editing site. For unedited editing sites the mean normalized coverage for each position is reported. For editing sites were edited bases were observed in the mRNA-seq data, the normalized coverage was multiplied by the respective editing rate (giving higher impact to more highly edited sites) and averaged over this weighted normalized signals. For the position-specific analysis the following parameters were changed: all transcripts were used (irrespective of editing sites in close vicinity), the ribosome profiling signal was normalized by the total signal in the ±1000 nt range, and transcripts were grouped according to the position of the editing site in the codon.

## RESULTS

### An *in vitro* translation system to systematically test the decoding of inosines

**Figure 2.**
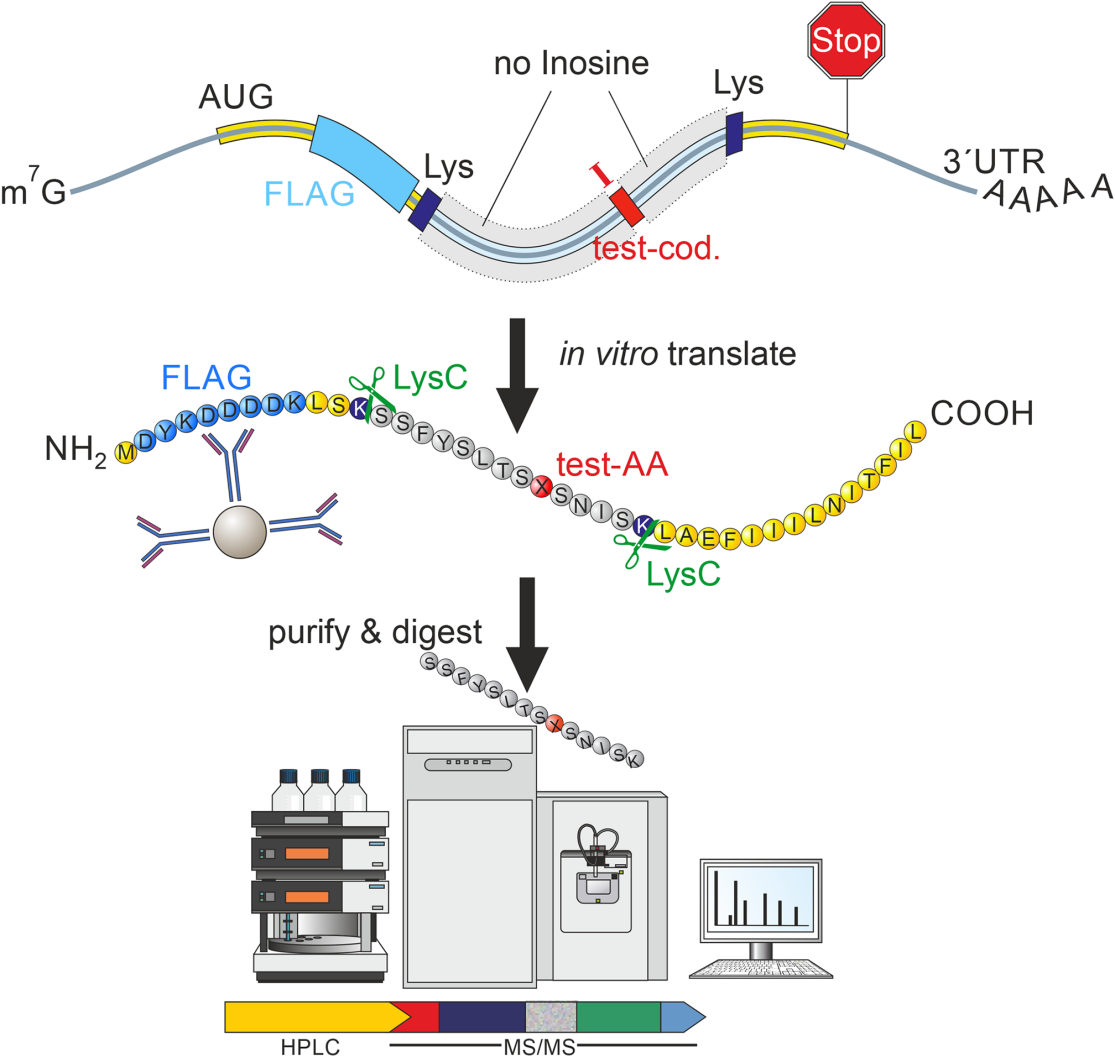
A setup to systematically test for the decoding of inosines in mRNA. A DNA template was designed that allows the *in vitro* transcription of an RNA containing a single inosine containing codon (red). The RNA also encodes a FLAG tag (light blue) and two lysine codons (dark blue). Upon *in vitro* translation using rabbit reticulocyte lysate the short protein can be purified using anti-FLAG antibodies. Following cleavage of the translated protein with LysC the peptide containing a single test amino acid (AA, red) is identified by mass spectrometry.

**Figure 3.**
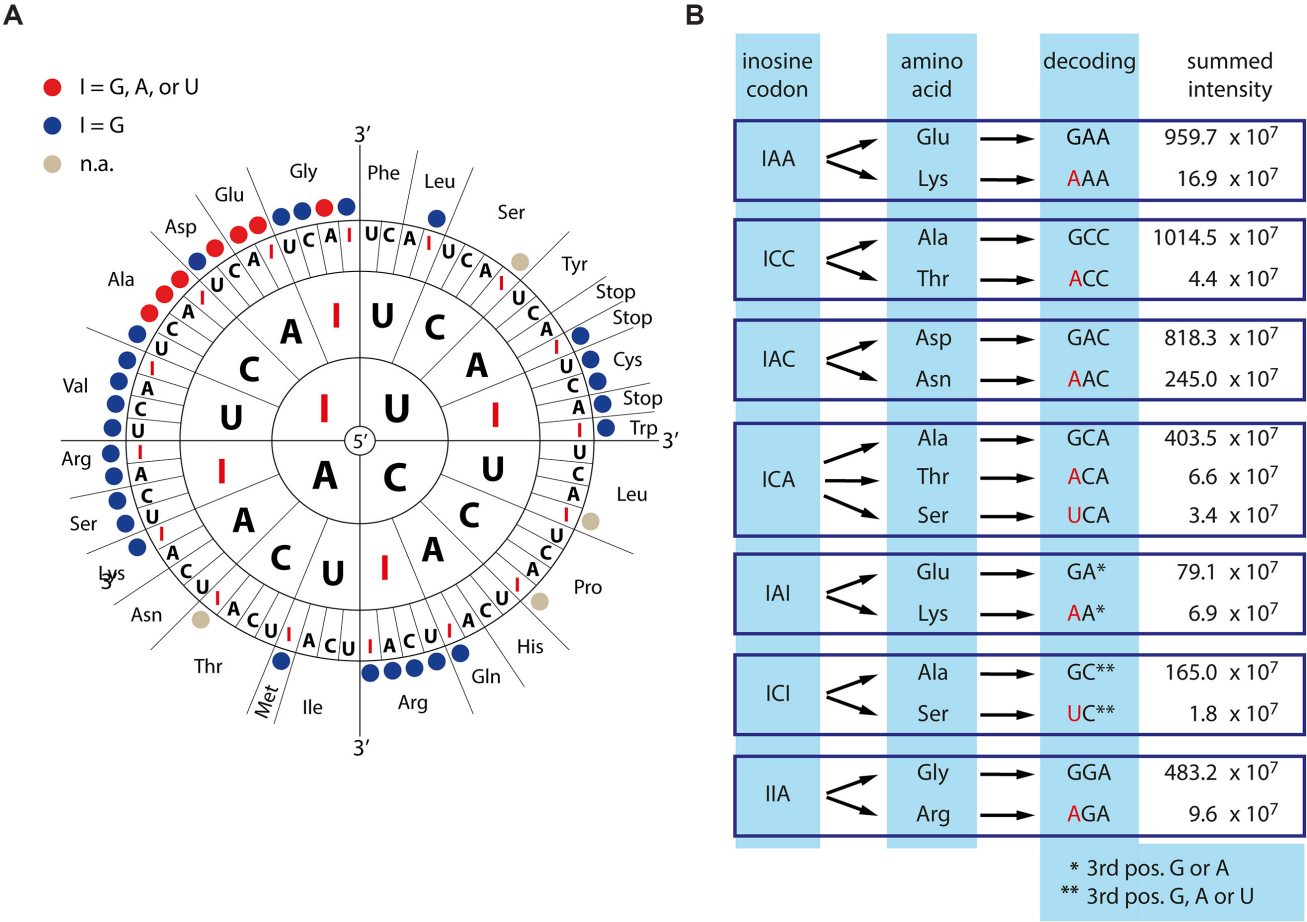
Context-dependent decoding of inosines as guanosine, adenosine, or uracil. (**A**) Scheme showing all codons where substitution of G by I may lead to recoding. For multiple codons peptide variants were detected that support decoding of inosine as adenosine or uracil (I = G, A, or U; A or U >0.4%; red dots). Codons giving rise to ambiguous products due to redundancy of the genetic code were omitted (n.a.; gray dots). Codons where only guanosine-decoding was detected are marked by a blue dot. (**B**) The peak intensities of the detected peptides supporting primary and alternative decoding are given (see Supplementary Table S1 for all tested codons). Alternative decoding is highlighted in red. Two test-codons contain inosine in the wobble position (marked by * or **). As either *A/G or **A/G/U would result in the same amino acid we cannot make a statement of the decoding at this position.

Inosines in mRNAs are generally believed to be decoded as guanosines during translation. However, to systematically test how inosines in mRNAs are decoded and to quantify their base-pairing preferences with uracil or adenosine in tRNAs, we synthesized short reporter-transcripts coding for an N-terminal Flag-tag and a reporter-peptide containing inosine in a single, defined codon (Figure 2). The reporter transcript was *in vitro* translated and purified using an anti-Flag antibody. Subsequently, the affinity-purified peptide-pool was submitted to mass spectrometry. The detected amino acid encoded by the inosine-containing test-codon served as a read-out for the decoding (Figure 2). To exemplify, for the test-codon IAA, the amino acid glutamate is expected as IAA is thought to be decoded as GAA. If the amino acid lysine was detected in addition, this would indicate additional decoding of IAA as AAA. The reporter-transcripts were generated using *in vitro* transcription in the presence of C, U, A and I. Therefore, a reporter-transcript sequence was designed that only contained codons consisting of A, C, and U. Only the test-codon contained inosines. Using this reporter transcript, we tested all codons containing inosines in at least one position (Figure 3). Codons that would lead to ambiguous translation products (mostly codons with inosines in the wobble position) were omitted.

To test whether T7 RNA polymerase would faithfully incorporate I instead of a G during preparation of the *in vitro* transcribed reporter-transcripts, we performed Illumina-Sequencing of cDNA prepared from the reporter-transcripts. Illumina-Sequencing revealed a high fidelity rate at the test-codon (>99.8%). The error rate of 0.2% is comparable with the overall error rates in NGS sequencing (49) (Supplementary Figure S1). This indicates faithful incorporation of inosine at the test codon by T7 RNA polymerase but also the correct incorporation of C, opposite the inosine during cDNA synthesis. Further, no significant misincorporation was observed at any other position, suggesting that inosine was not incorporated at any other position than the test codon.

### The translation machinery decodes inosine as guanosine, adenosine or uracil

Test-transcripts were *in vitro* translated using rabbit reticulocyte lysate. The peptide sequence was designed to be flanked by two lysine-codons that were used to digest the translated protein by Lysyl endopeptidase (Lys-C) prior to mass spectrometric analysis (Figure 2). The resulting peptides were analyzed by LC–MS/MS. Mass spectrometry spectra were generated using MaxQuant (40). Representative spectra for all peptides are shown (Supplementary Data S1). For quantification of the spectra intensities all spectra were carefully inspected using Skyline and manually extracted (41) (Supplementary Table S1). Only peptides supporting alternative decoding observed at percentages >0.4% compared to the expected peptide were considered for further analysis. Interestingly, 7 out of 33 codons exhibited unexpected decoding (Figure 3A). While inosine was interpreted as guanosine in all codons, in five codons inosine was also decoded as adenosine (Figure 3B). In one codon inosine was decoded as both adenosine and uracil, while one codon exhibited alternative decoding as uracil. Alternative decoding was restricted to position 1 of the codon.

### Alternative decoding is particularly high for IAC and IAI

**Figure 4.**
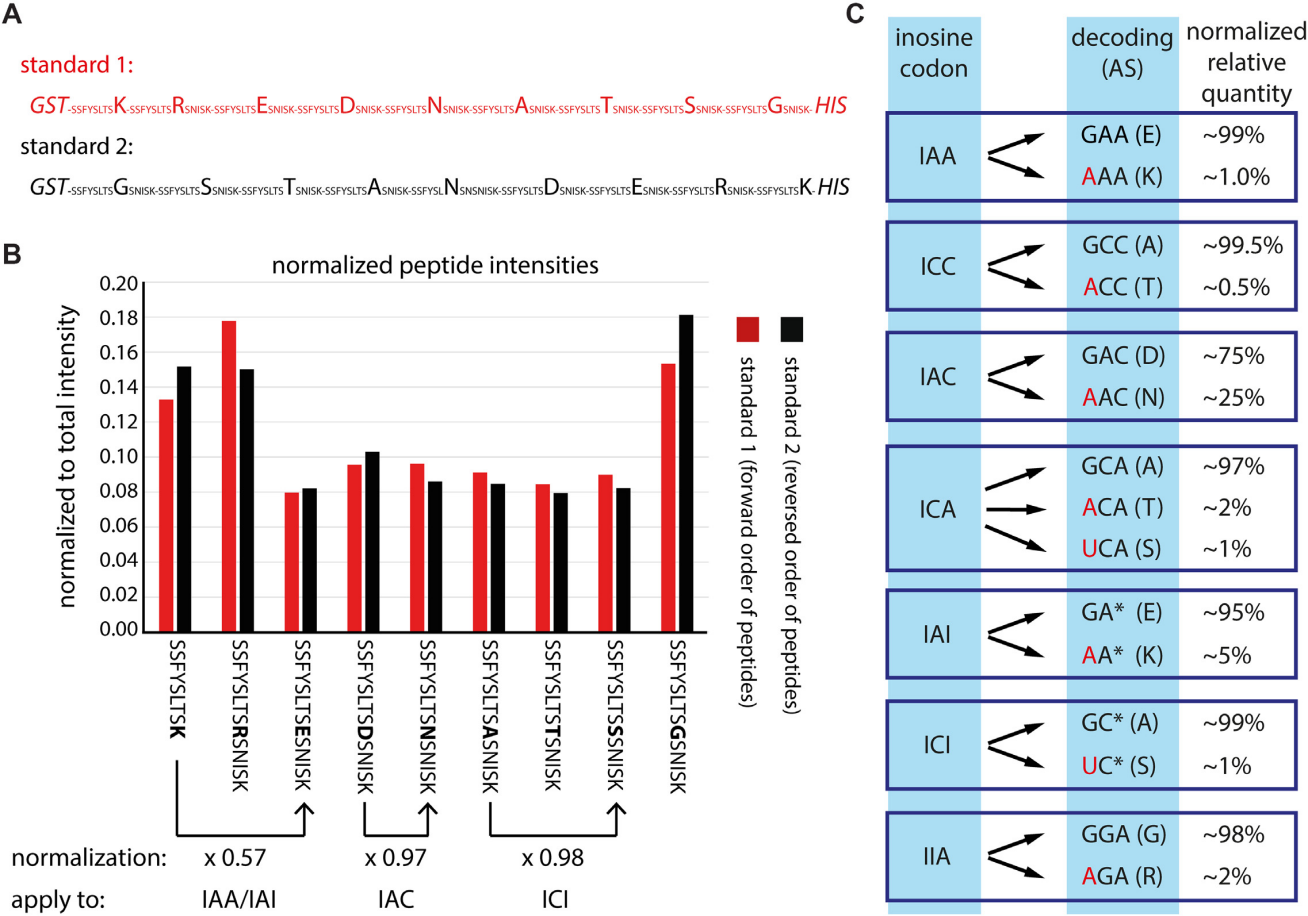
Alternative decoding is significant for codons IAC and IAI. (**A**) For normalization of mass spectrometry results, all peptides with substantial alternative decoding were expressed as one concatenated peptide in two orders (forward: red, reverse: black) in *E. coli* with an N-terminal GST-tag and a C-terminal His-tag. The peptide sequence is given and changed amino acids are highlighted. (**B**) After expression and purification the individual peptides were released using Lys-C and measured by mass spectrometry. The individual peptide intensities were normalized to the summed total intensity. The peptide sequence is given below. For differences in intensity between two peptides a normalization factor (normalization) is calculated based on the average intensity between standard 1 and standard 2. Three examples are given below the peptide sequence. The factors are then applied to the peptide intensities detected after *in vitro* translation shown in: (**C**) The relative primary and alternative decoding after normalization are given. Red indicates alternative decoding. The detected amino acid is shown in brackets.

Mass spectral intensities for different peptides do not always reflect the true amount of the peptide. To allow for a normalization of potential differences in the detection of peptides, all peptide permutations (i.e. peptides supporting alternative decoding and the peptide supporting expected decoding) were recombinantly expressed as a single, concatenated fusion protein in *E. coli*. The fusion protein carried an N-terminal GST-tag and a C-terminal 6xHis-tag. To ensure that full-length protein is produced, the fusion proteins were generated in two different versions (standard 1 and standard 2) with peptides in different orders (Figure 4A). Following separation of the induced protein by SDS-PAGE, the expressed fusion proteins were excised from the gel, digested, and submitted to mass spectrometry. Importantly, all peptides in standard 1 and standard 2 exhibited similar intensities irrespective of their position in the protein indicating no strong bias in their mass spectrometric detection (Figure 4B). Subsequently, the mean of the intensities for standard 1 and standard 2 were used to normalize intensities of the *in vitro* translated peptides. For instance, the intensity for the peptide containing a K is approximately 2-times stronger than for the peptide containing an E (Figure 4B). This is relevant for the inosine codons IAA and IAI as they both yield peptides with K or E supporting decoding as A or G. The normalization was done accordingly for all *in vitro* translation products. After normalization alternative decoding ranging between 0.5% and 2% was detected for 5 codons (Figure 4C). Much higher decoding of inosine as adenosine was observed for the two codons IAC (25%) and IAI (5%).

We also tested if inosine could replace the guanosine in STOP-codons. For UIA (UGA) and UAI (UAG) a peptide supporting accurate translational termination was detected (Supplementary Table S1). For UIA no translational read-through was detected whereas for UAI about 1–2% full-length peptide – containing Tyr at the position of the STOP-codon – was detected. This suggests that UAI was interpreted as UAU in this case.

### Inosine induces ribosome stalling

**Figure 5.**
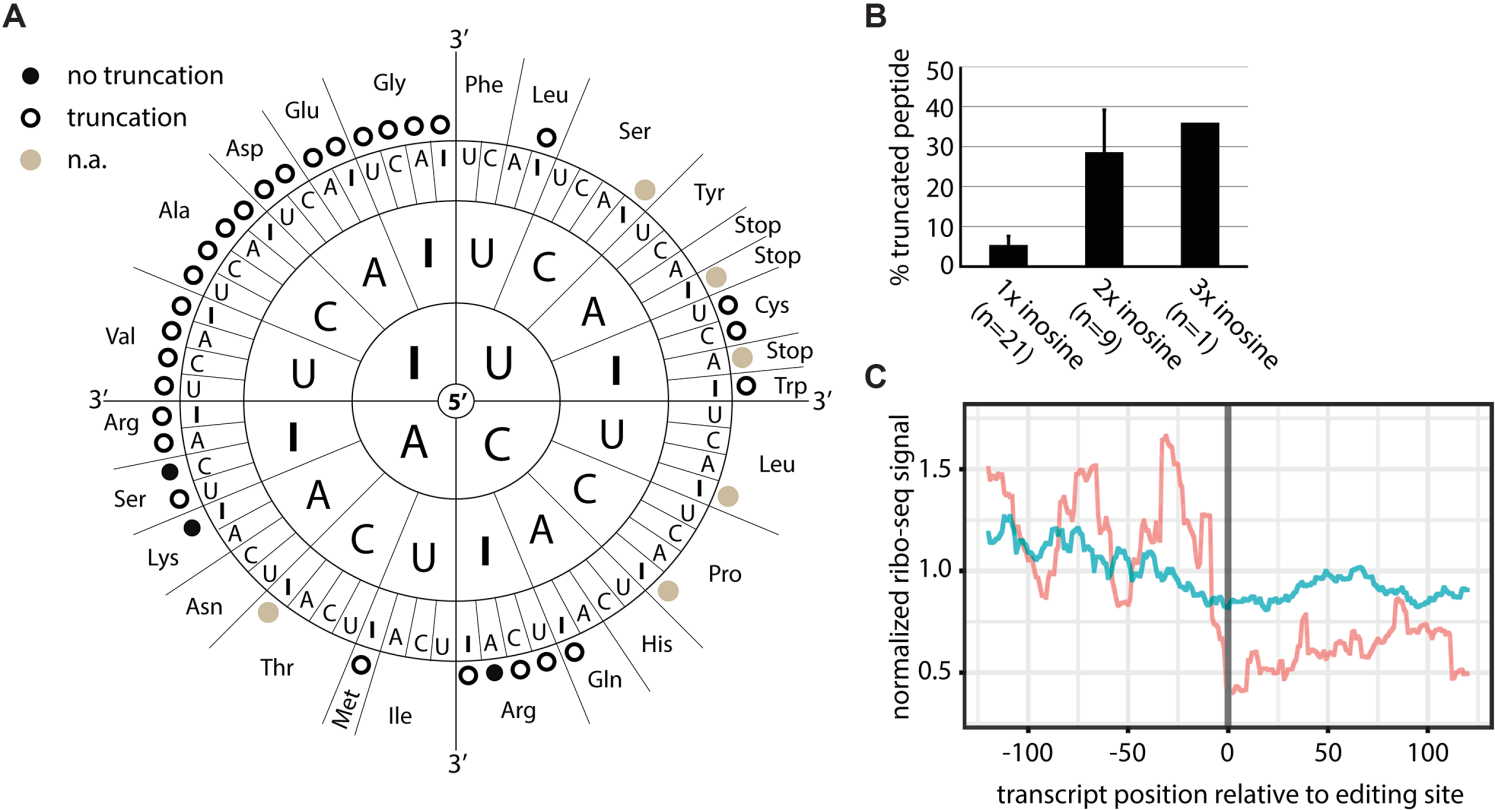
Inosine causes ribosome stalling. (**A**) Black dots indicate exclusive detection of full-length peptide, circles indicate additional truncated peptides. Codons with inosine only in the wobble position or STOP codons were omitted (n.a.; gray dots). (**B**) Percentage of truncated peptide detected for different numbers of inosines in the codon are shown. Error bars = s.e.m. (**C**) Inosine levels at known editing sites were calculated from brain mRNA-seq data. Ribosome profiling data for sites showing editing (red) or no editing (blue) were normalized, weighted by editing rate, merged, and centered on the editing site. The coverage from position −125 to +125 relative to the editing site is given.

Interestingly, 28 out of the 31 tested codons (excluding STOP-codons) gave rise to a truncated peptide (Figure 5A). Some of those also exhibited alternative decoding. The peptide was found truncated immediately upstream of the test-codon position. This suggested ribosome stalling which can be precisely assigned to particular codons (50). We detected truncated peptides for all codons with two or more inosines while only 18 out of 21 codons with only a single inosine led to truncations (Figure 5A, Table 1). Also, truncation rates varied with the number of inosines present. Codons with a single inosine led to lower percentage of truncations (∼5%), while codons with two or three inosines lead to substantial truncation rates of about 30% (Figure 5B). Especially codons with inosine both in the first and last position lead to high truncation rates (III, IAI, ICI, IUI) (Table 1). Finally, all codons with inosine in position 1, and most codons with inosine in position 2 or 3 led to the formation of truncated peptides. However, the average truncation rate across all possible inosine containing codons showed no strong position-specific bias.

**Table 1.** Peptide truncation is strongest when multiple inosines occur in the codon. The percent truncation (>0.4%) is grouped according to the number of inosines per codon (left) or the position of a single inosine in the codon (right)

1× inosine		2× inosine		3× inosine		First position		Second position		Third position	
UUI	49%	IAI	84%	III	36%	IUA	5%	AIA	7%	UUI	49%
AIA	7%	ICI	64%			IAA	5%	UIU	6%	AUI	3%
UIU	6%	UII	57%			ICA	3%	UIC	5%	CAI	3%
IUA	5%	IUI	26%			IAU	3%	CIC	4%	AAI	0%
IAA	5%	CII	8%			ICC	3%	AIU	3%		
UIC	5%	IIA	7%			IUC	3%	CIU	3%		
CIC	4%	AII	4%			ICU	2%	CIA	0%		
ICA	3%	IIU	4%			IUU	2%	AIC	0%		
AUI	3%	IIC	2%			IAC	1%				
AIU	3%										
CAI	3%										
IAU	3%										
ICC	3%										
IUC	3%										
CIU	3%										
ICU	2%										
IUU	2%										
IAC	1%										
AAI	0%										
CIA	0%										
AIC	0%										

To see whether inosine can also affect translation *in vivo*, we analyzed publicly available ribosome-profiling and mRNA-seq data that were available at high quality from human brain tissues (51). We determined editing levels at known human editing sites (46) using the available, corresponding mRNA-seq data. Subsequently, we classified all editing sites as either edited or unedited. To ensure that an edited site next to an un-edited site would not skew the ribosome-profiling data, all transcripts with editing sites in close vicinity (±5 nts) were removed. Subsequently, meta-plots of corresponding ribosome profiling data were generated by overlaying coverage, weighted by editing rates, of all edited transcripts centered on the editing site (Figure 5C). Coverage dropped at editing sites, indicating that inosines in the decoding center of the ribosome affect translation kinetics, leading to ribosome stalling. Unedited transcripts used as a control showed no drop in ribosome density (Figure 5C).

**Figure 6.**
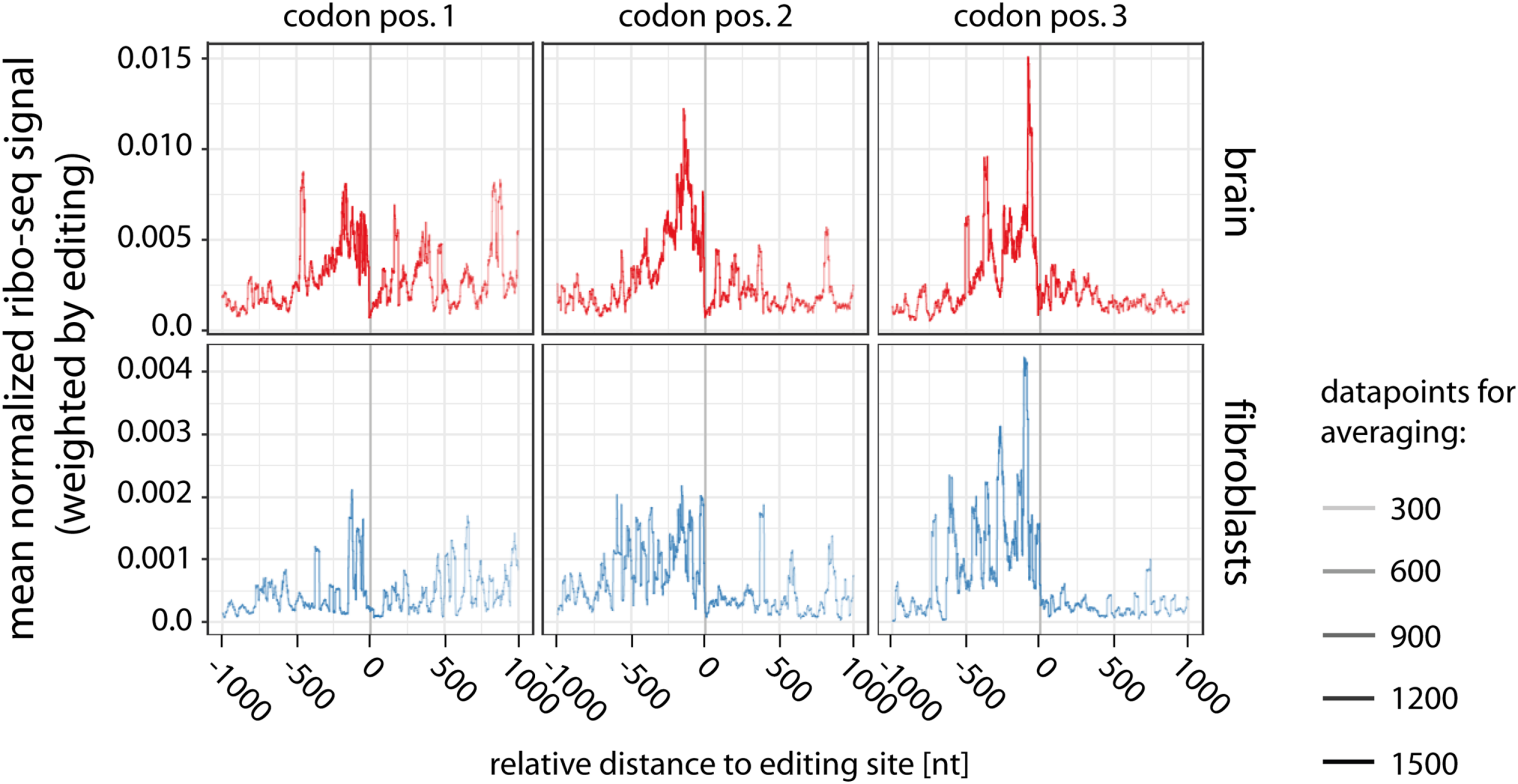
Inosine-induced ribosome stalling is position-independent and occurs in multiple tissues. Inosine levels at known editing sites were calculated from brain mRNA-seq data (top). Ribosome profiling data (ribo-seq) were normalized, weighted by editing rate, merged, and centered on the editing site split according to the position of the inosine in the codon (codon pos.1–3). The coverage from position −1000 to +1000 relative−to the editing site is given. For comparison, ribosome-profiling data from interferon-induced fibroblasts was analyzed (bottom). The number of data points used for averaging is reflected by the thickness of the line.

To determine if the position of the editing site in the codon was relevant, ribosome-profiling data were split according to the position of the editing site in the codon. To include as many transcripts as possible into this analysis we also included transcripts with editing sites in close vicinity. Again, coverage dropped at the editing site (Figure 6, top). The effect was similar irrespective of the position of the inosine in the codon. To substantiate this finding, the analysis was repeated with a ribosome-profiling dataset from interferon-stimulated fibroblasts (52). Also here, a position-independent drop in ribosome coverage was observed at the position of detected editing events (Figure 6, bottom). In conjunction with the *in vitro* translation data, the ribosome-profiling data suggest inosine-induced stalling of ribosomes, independent of the position of the edited nucleotide within the codon.

## DISCUSSION

Using a model mRNA that allows to systematically test the decoding of inosine containing codons, we show that inosine can be decoded as guanosine, adenosine, and uracil. This is in contrast to the prevalent view where inosine in mRNAs is generally believed to be decoded as a guanosine. The *in vitro* translation system used here, shows that position 1 of the codon is most sensitive to the presence of inosines. This is reminiscent of previous findings showing that position 1 of a codon is also most sensitive to the presence of pseudouridine and suggests that position 1 of a codon might in general be most sensitive to chemical modifications (34,53).

The generally accepted notion in the field that inosine is translated as guanosine originates from two key observations: Firstly, mice lacking the ADAR2 editase responsible for deaminating the glutamate receptor *Gria2* pre-mRNA die 2–3 weeks after birth. These mice can be rescued when a *Gria2* allele coding for a guanosine instead of an adenosine is introduced (24). However, this study gives limited insight to the general decoding of inosines as the decoding of inosine could be codon-specific. Secondly, using purified *E. coli* ribosomes a polymer of U and I nucleotides in a 5:1 ratio led to similar amino acid incorporation rates as did a polymer consisting of U and G nucleotides (29). Obviously, our understanding of ribosome decoding and translation efficiency has dramatically improved over the last 5–6 decades. Therefore, revisiting the problem using a state-of-the-art, unbiased approach seemed appropriate. Indeed, we could show that inosine is not only decoded as guanosine but also as A or U, in a codon-dependent manner.

At present, our findings are limited to an *in vitro* translation assay that used a particular reporter sequence throughout. Therefore, further studies will be required to appreciate the impact of inosine recoding *in vivo*. In any case, the fact that inosines can be decoded in multiple ways suggests that A-to-I RNA editing can lead to complex changes at the protein level. For instance, our study shows that IAC can be decoded to 75% as Asp and to 25% as Asn. Therefore, if, for instance, the first base of an AAC codon is edited to 50% *in vivo*, still only 37.5% of proteins would contain Asp (GAC) while 62.5% would still contain an Asn (AAC) at the corresponding position. However, for the majority of codons the level of editing seems closely reflected at the protein level. It is also worth noting, that variable decoding was only observed at the test codon and not at any other position. This indicates that T7 RNA polymerase incorporated inosine exclusively at the test codon but not at other positions, at least not at rates that would be detectable.

Detailed understanding of how editing events introduced in RNA are reflected at the protein level will also be of importance when developing (therapeutic) site-directed editing approaches where ADAR-deaminase activity is directed to a particular adenosine in order to correct aberrant protein functions (54–58). For these approaches, it will be important to understand that the consequence of an artificially introduced RNA-editing event will strongly depend on the context and the position of the introduced inosines within the codon.

Most surprisingly, our data strongly suggest that inosines can induce ribosome stalling, both *in vitro* and *in vivo*. Our analysis detects peptides that are truncated immediately upstream of the inosine-containing codon. Similar findings were made for RNAs containing N1-methyl-pseudouridine, which also gave rise to truncated proteins, most likely due to ribosome-stalling (59). Our analysis of ribosome-profiling data suggest further that stalling of ribosomes starts about 400–500 nts upstream of the editing site (Figure 6). This is in rough agreement with previous observations suggesting that up to nine ribosomes accumulate before a ribosome stalling site (50). Our observation that multiple inosines in an mRNA strongly impair translation may be of relevance for transcripts containing consecutive editing sites. For instance, the transcript encoding serotonin receptor HTR2C harbors five editing sites that are closely spaced in a window of only 13 nucleotides (60,61). It will be interesting to test how the editing state of the HTR2C transcript is reflected at the protein level and whether hyperediting can lead to the formation of truncated proteins.

While we observed that all tested codons with inosine in the first position led to truncated peptides during *in vitro* translation, the average rate of truncation was relatively low. In contrast, the highest rate of a truncated peptide was observed for a single codon carrying inosine in position 3. Again, calculated over all possible codons carrying inosine in position 3, the number of detectable truncated peptides would be low. Thus, on average, the rate of detectable truncated peptides is comparable for all positions of the codon. In agreement with this, we did not detect any position-specific effect for ribosome stalling in the ribosome profiling data. Moreover, one needs to consider, that the RNAs tested by *in vitro* translation were artificially generated. *In vivo* editing by ADAR shows a strong bias, introduced by the next-neighbor nucleotide preferences of the catalytic domains of ADAR1 and ADAR2 (62). Therefore, the ribosome profiling data will not contain all possible combinations that were generated *in vitro*.

**Figure 7.**
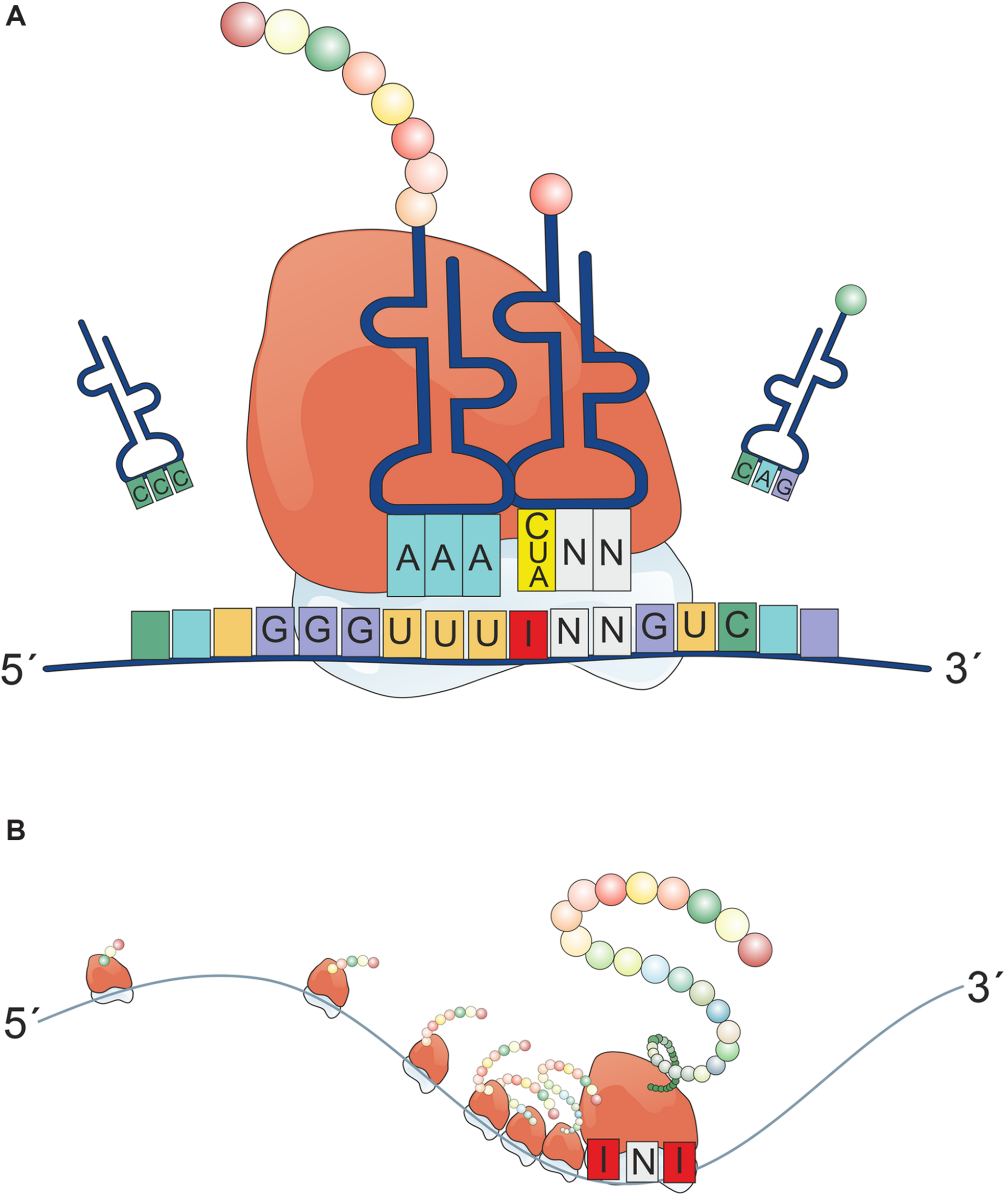
Inosines in mRNAs can lead to different recoding events and promote ribosome stalling. (**A**) A single inosine in a codon of an mRNA can basepair with C, U, or A in the corresponding tRNA, leading to different decoding events. (**B**) Especially the presence of multiple inosines but also individual inosines in a codon seemingly provokes ribosome stalling and the formation of truncated peptides.

Today, an increasing body of literature demonstrates that RNA-modifications directly or indirectly influence translation. For instance, N1-methyl-pseudouridine increases the rate of translation by turning off eIF2α–phosphorylation-mediated inhibition of translation, but also by increasing ribosome-density on mRNA (59). 5-methylcytosine can lead to alternative decoding of mRNAs (63). Pseudouridylation can suppress STOP-codons (64). N1-methyladenosine is positively correlated with elevated protein expression and clustered around the start codon suggesting a potential role during translation initiation (65). Here, we have shown that also inosine modulates translation to a previously unappreciated extent. In sum, our data shows that the presence of inosines in mRNAs can increase the coding potential of an mRNA in more than one way and affect translation dynamics at the same time (Figure 7).

## DATA AVAILABILITY

MaxQuant software package (http://www.maxquant.org/); uniprot database (www.uniprot.org); Bam-readcount (https://github.com/genome/bam-readcount).

The mass spectrometry proteomics data have been deposited to the ProteomeXchange Consortium via the PRIDE partner repository with the dataset identifier PXD010329. Ribosome profiling (SRR1562539, SRR1562540, and SRR1562541) and mRNA-seq (SRR1562544, SRR1562545, and SRR1562546) data were obtained from the short read archive (SRA).

## Supplementary Material

Supplementary Data
