# Supplementary material for: Inosine induces context-dependent recoding and translational stalling
Source: Nucleic Acids Res. 2018 Nov 20;47(1):3–14. doi: 10.1093/nar/gky1163 (PMC6326813; doi:10.1093/nar/gky1163)
Supplement: Supplementary Data [file gky1163_supplemental_files.pdf]

Supplementary Information

Inosine induces context-dependent recoding and translational stalling

Konstantin Licht, Markus Hartl, Fabian Amman, Dorothea Anrather, Michael P. Janisiw, Michael F. Jantsch

Table of Contents

Supplementary Figure S1: page 2

Supplementary Table S1: page 3-5

Supplementary Dataset S1: page 6-23

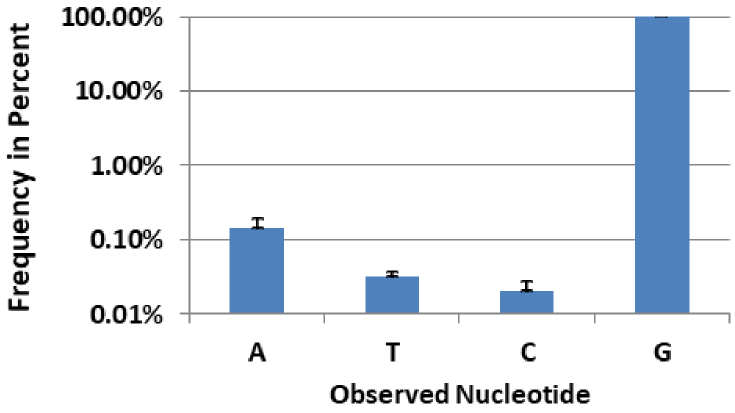

Supplementary Figure S1. The fidelity of *in vitro* transcription with inosine is very high. Using RT-PCR and amplicon-seq the rate of mis-incorporation of other nucleotides instead of inosine was tested. The frequency of all nucleotides detected at positions with inosine is given. n=11, error bars = s.e.m.

Supplementary Table S1. All measured peak intensities for the detected peptides after *in vitro* translation, affinity purification, and mass spectrometry of inosine-containing codons. Sample#: number of sample, reflects also the order of measurement. Codon: inosine-containing codon. Decoding/Interpretation: Codon (either A, U, G), truncation (truncated peptide), STOP (Stop-codon), carryover (in 3 samples a peptide from the previous run was detected. For these three samples we have analyzed the wash runs between samples and could detect the peptide-signal with decreasing intensities, strongly arguing for carryover). Peptide: peptides detected. Summed peak intensities (log2) using Skyline (the summed intensities of all fragments that could be assigned to the respective peptide, log2).

Sample#	Codon	Decoding/ Interpretation	Peptide	Summed peak intensities (log2) using Skyline	Remarks
1	AIC	AGC	SSFYSLTS**S**SNISK	32.34	
2	CIA	CGA	SSFYSLTS**R**SNISK	32.26	
3	CII	CGG	SSFYSLTS**R**SNISK	34.49	
	CII	truncation	SSFYSLTS*	30.88	
4	UIA	STOP	SSFYSLTS*	29.2	
5	IUA	GUA	SSFYSLTS**V**SNISK	33.62	
	IUA	truncation	SSFYSLTS*	29.49	
6	IAA	GAA	SSFYSLTS**E**SNISK	33.16	
	IAA	AAA	SSFYSLTS**K**	27.33	
	IAA	truncation	SSFYSLTS*	29.01	
7	ICC	GCC	SSFYSLTS**A**SNISK	33.24	
	ICC	ACC	SSFYSLTS**T**SNISK	25.39	
	ICC	truncation	SSFYSLTS*	28.06	
8	IAU	GAU	SSFYSLTS**D**SNISK	34.23	
	IAU	truncation	SSFYSLTS*	29.09	
9	ICU	GCU	SSFYSLTS**A**SNISK	33.78	
	ICU	ACU	SSFYSLTS**T**SNISK	24.54	
	ICU	truncation	SSFYSLTS*	28.44	
10	AII	AGG	SSFYSLTS**R**SNISK	33.97	

	AII	truncation	SSFYSLTS*	29.5	
11	IUU	GUU	SSFYSLTSVSNISK	33.44	
	IUU	truncation	SSFYSLTS*	28.02	
12	IAC	GAC	SSFYSLTSDSNISK	32.93	
	IAC	AAC	SSFYSLTSNSNISK	31.19	
	IAC	truncation	SSFYSLTS*	27.08	
13	UAI	STOP	SSFYSLTS*	29.63	
	UAI	UAU	SSFYSLTSYSNISK	23.73	
14	UII	UGG	SSFYSLTSWSNISK	27.8	
	UII	truncation	SSFYSLTS*	28.23	
15	IUI	GUG	SSFYSLTSVSNISK	31.57	
	IUI	truncation	SSFYSLTS*	30.05	
16	AAI	AAG	SSFYSLTSK	35.87	
	AAI	truncation	SSFYSLTS*	27.83	
17	CIC	CGC	SSFYSLTSRSNISK	33.26	
	CIC	truncation	SSFYSLTS*	28.72	
	CIC	truncation	SSFYSLTSR	29.13	
	CIC	*carryover*	SSFYSLTSK	25.73	carryover from sample 16
18	AIU	AGU	SSFYSLTSSSNISK	32.57	
	AIU	truncation	SSFYSLTS*	27.65	
	AIU	*carryover*	SSFYSLTSK	23.73	carryover from sample 16
19	IUC	GUC	SSFYSLTSVSNISK	31.53	
	IUC	truncation	SSFYSLTS*	26.35	
20	ICA	GCA	SSFYSLTSASNISK	31.91	
	ICA	ACA	SSFYSLTSTSNISK	25.97	
	ICA	UCA	SSFYSLTSSSNISK	25.03	
	ICA	truncation	SSFYSLTS*	27.16	
21	IIU	GGU	SSFYSLTSGSNISK	31.19	
	IIU	truncation	SSFYSLTS*	26.71	
22	IAI	GAG	SSFYSLTSESNISK	29.56	
	IAI	AAG/AAA	SSFYSLTSK	26.03	
	IAI	truncation	SSFYSLTS*	32.08	

23	ICI	GCG	SSFYSLTSASNISK	30.62	
	ICI	UCG	SSFYSLTSSSNISK	24.11	
	ICI	truncation	SSFYSLTS*	31.46	
24	UIC	UGC	SSFYSLTSCSNISK	31.84	
	UIC	truncation	SSFYSLTS*	27.63	
25	UIU	UGU	SSFYSLTSCSNISK	30.23	
	UIU	truncation	SSFYSLTS*	26.35	
26	CAI	CAG	SSFYSLTSQSNISK	32.37	
	CAI	truncation	SSFYSLTS*	27.36	
27	IIC	GGC	SSFYSLTSGSNISK	32.22	
	IIC	truncation	SSFYSLTS*	26.36	
	IIC	*carryover*	SSFYSLTSQSNISK	22.93	carryover from sample 26
28	III	GGG	SSFYSLTSGSNISK	33.08	
	III	truncation	SSFYSLTS*	32.25	
29	CIU	CGU	SSFYSLTSRSNISK	30.99	
	CIU	truncation	SSFYSLTS*	25.73	
30	AIA	AGA	SSFYSLTSRSNISK	29.39	
	AIA	truncation	SSFYSLTS*	25.73	
31	AUI	AUG	SSFYSLTSMSNISK	31.83	
	AUI	truncation	SSFYSLTS*	27	
32	IIA	GGA	SSFYSLTSGSNISK	32.17	
	IIA	AGA	SSFYSLTSRSNISK	26.51	
	IIA	truncation	SSFYSLTS*	28.41	
33	UUI	UUG	SSFYSLTSLSNISK	26.8	
	UUI	truncation	SSFYSLTS*	26.72	

Supplementary Dataset S1. Representative spectra for all detected peptides. Spectra (taken from MaxQuant) are shown. If the same peptide occurred in multiple samples, one spectra was randomly chosen.

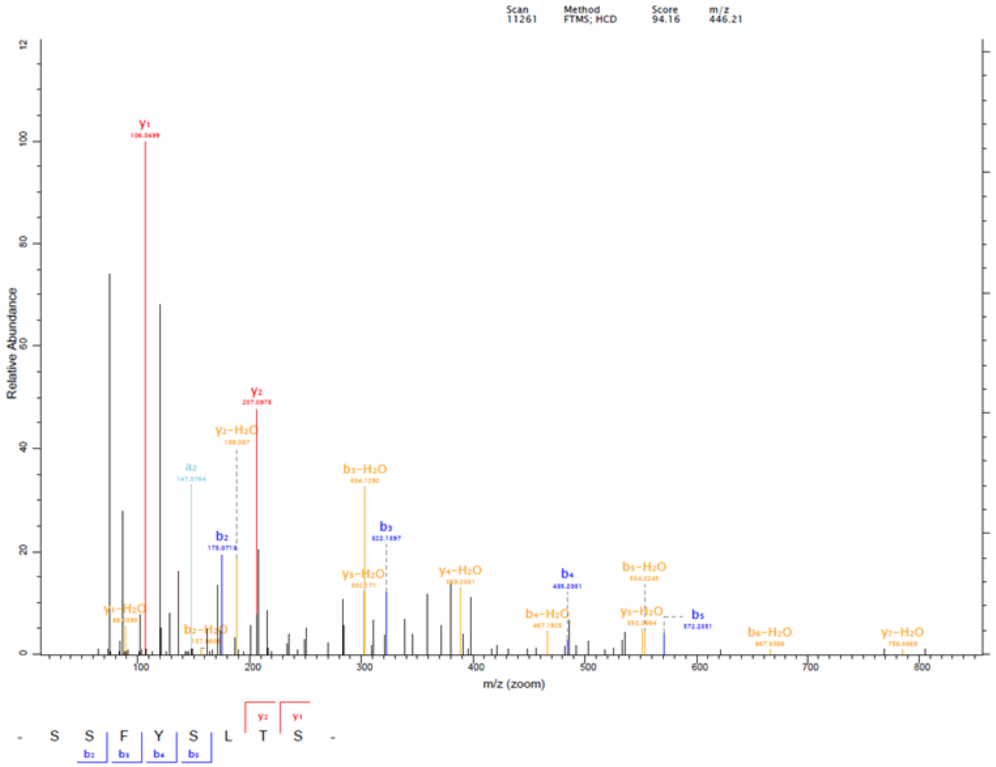

Scan 11261
Method FTMS; HCD
Score 94.16
m/z 446.21
Relative Abundance
m/z (zoom)
y1
y2
y1-H2O
y2-H2O
a2
b2
b2-H2O
b3-H2O
b3
y3-H2O
y4-H2O
b4-H2O
b4
b5-H2O
y5-H2O
b5
b6-H2O
y7-H2O
- S S F Y S L T S -

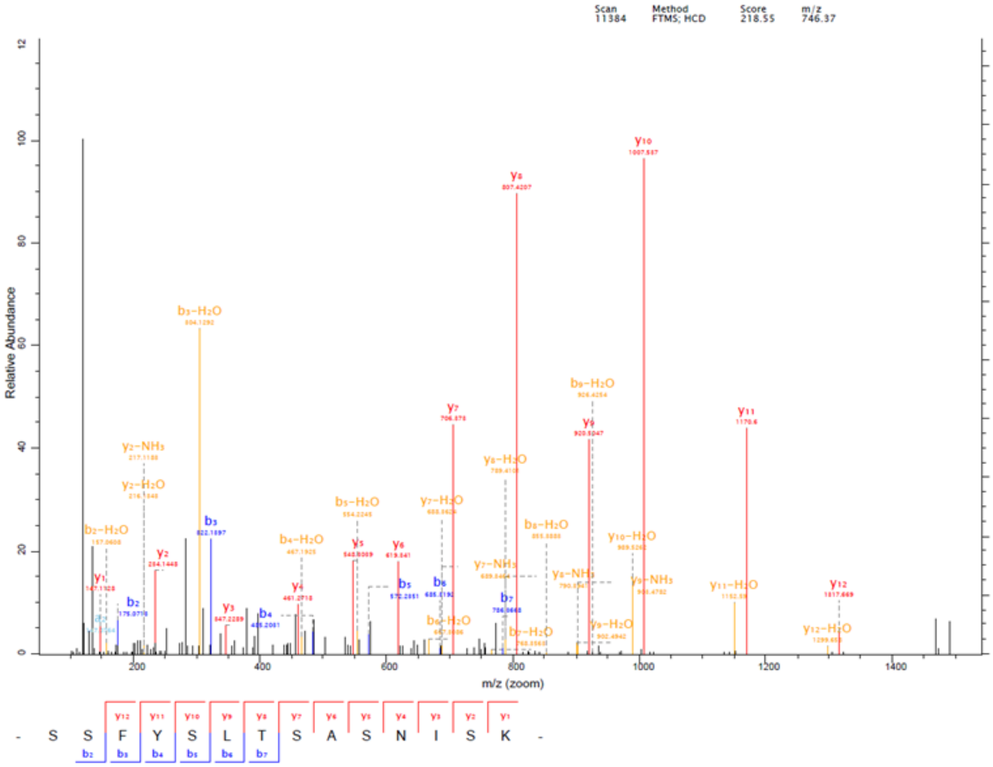
Scan 11384
Method FTMS; HCD
Score 218.55
m/z 746.37
Relative Abundance
m/z (zoom)
- S S F Y S L T S A S N I S K -

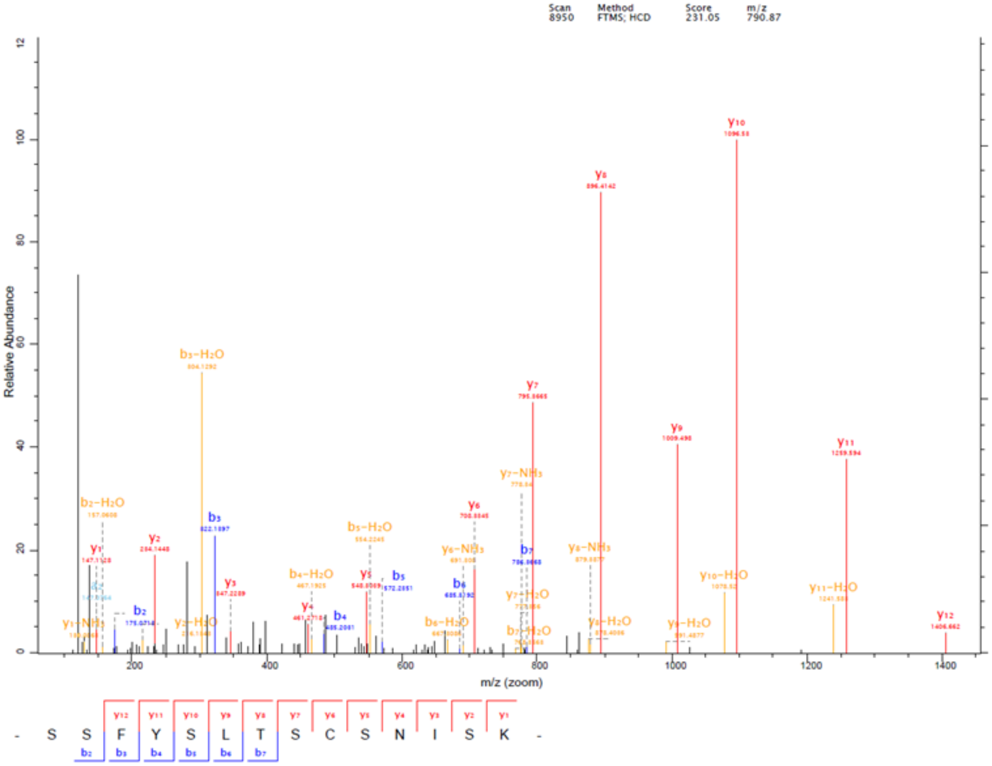
Scan 8950
Method FTMS; HCD
Score 231.05
m/z 790.87
Relative Abundance
m/z (zoom)
y10
y8
y7
y9
y11
y12
y6
y2
y1
y3
y4
y5
b3-H2O
b2-H2O
b3
b2
b4
b5
b6
b7
b4-H2O
b5-H2O
y7-NH3
y8-NH3
y10-H2O
y11-H2O
y7-H2O
b7-H2O
b6-H2O
S S F Y S L T S C S N I S K

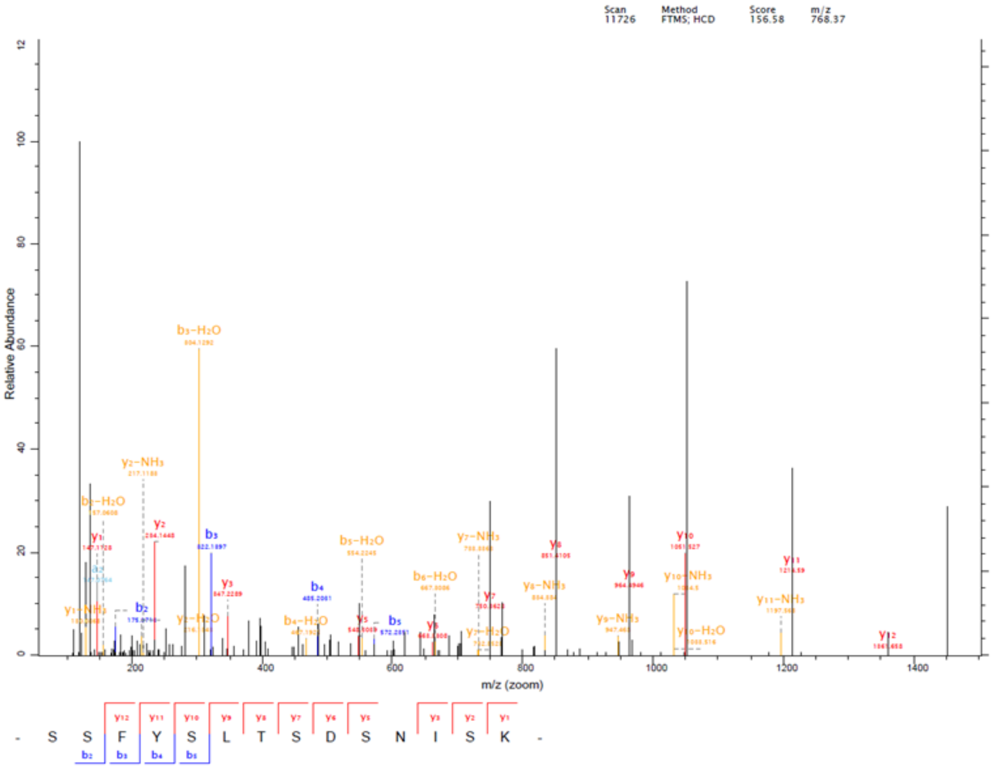
Scan 11726
Method FTMS; HCD
Score 156.58
m/z 768.37
Relative Abundance
m/z (zoom)
- S S F Y S L T S D S N I S K -

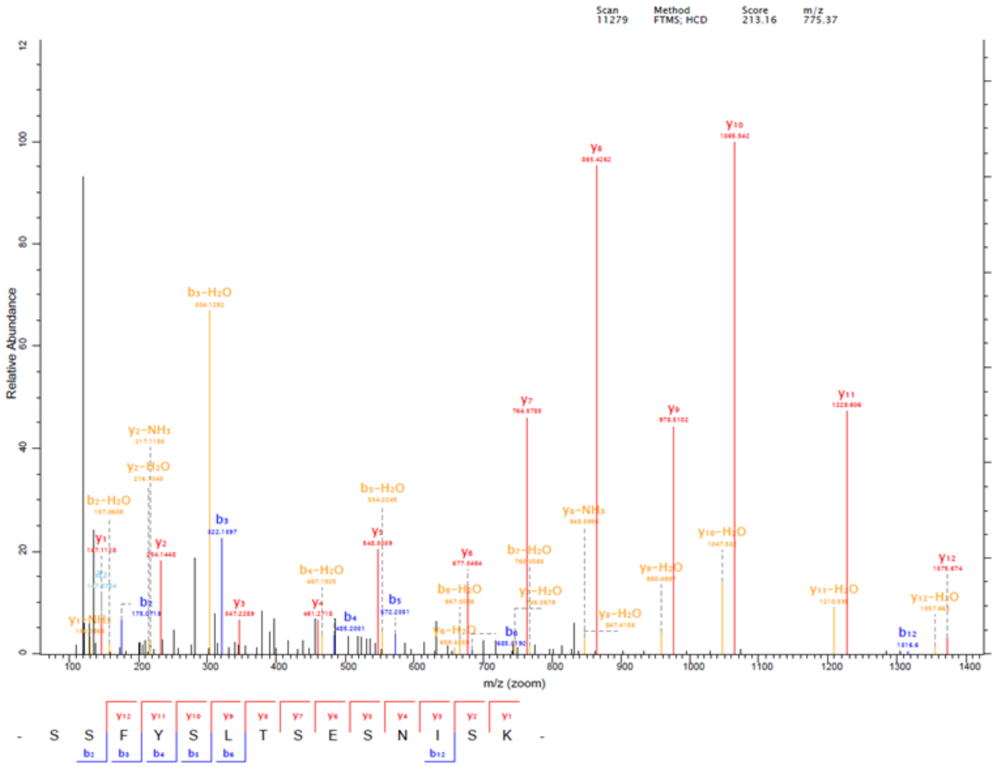
Scan
11279
Method
FTMS; HCD
Score
213.16
m/z
775.37
Relative Abundance
m/z (zoom)
- S S F Y S L T S E S N I S K -

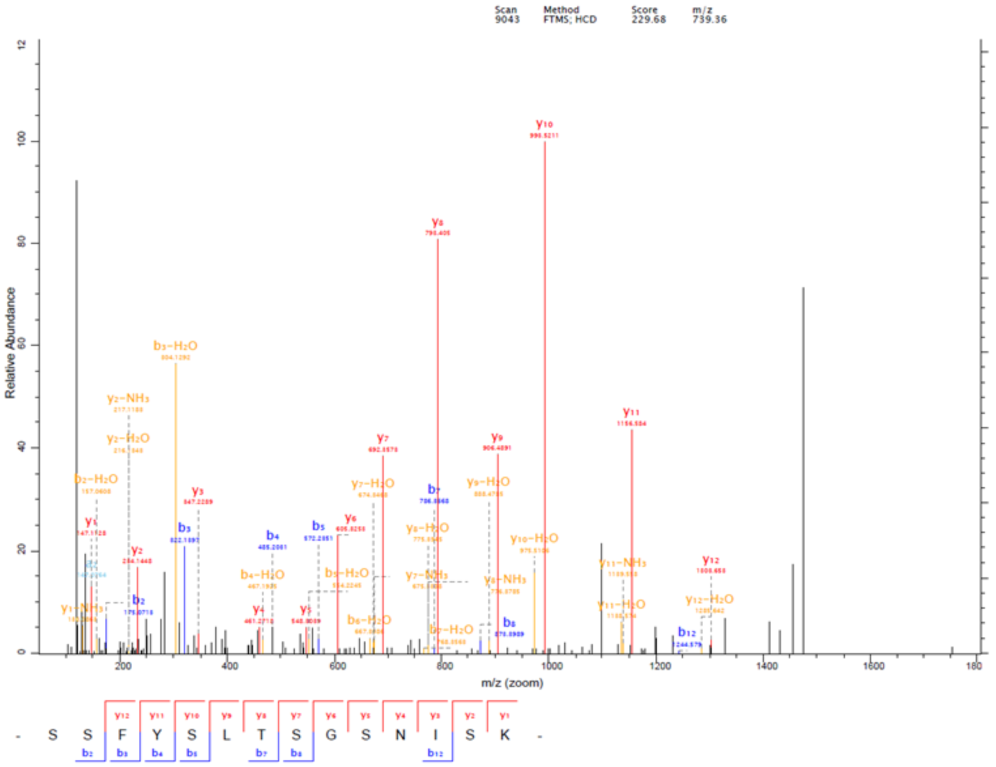
Scan 9043
Method FTMS; HCD
Score 229.68
m/z 739.36
Relative Abundance
m/z (zoom)
- S S F Y S L T S G S N I S K -

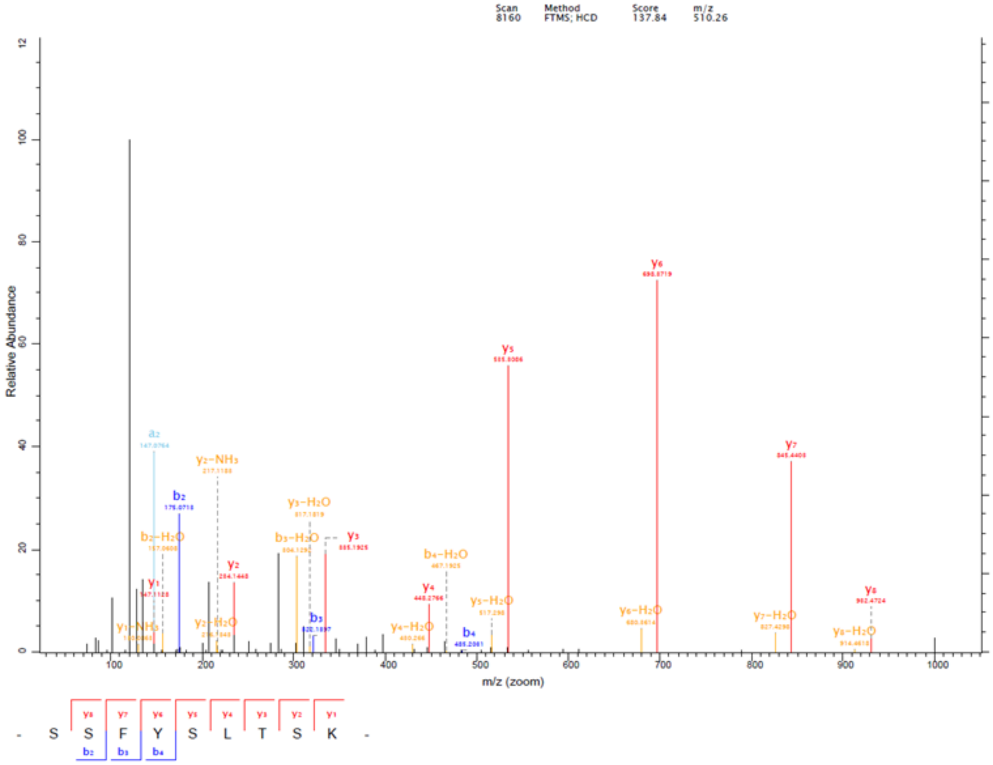

Scan 8160
Method FTMS; HCD
Score 137.84
m/z 510.26
Relative Abundance
m/z (zoom)
a2
b2
b2-H2O
y2-NH3
y1
y2
y3-H2O
b3-H2O
y3
b3
y4
b4-H2O
y4-H2O
b4
y5
y5-H2O
y6
y6-H2O
y7
y7-H2O
y8
y8-H2O
- S S F Y S L T S K -

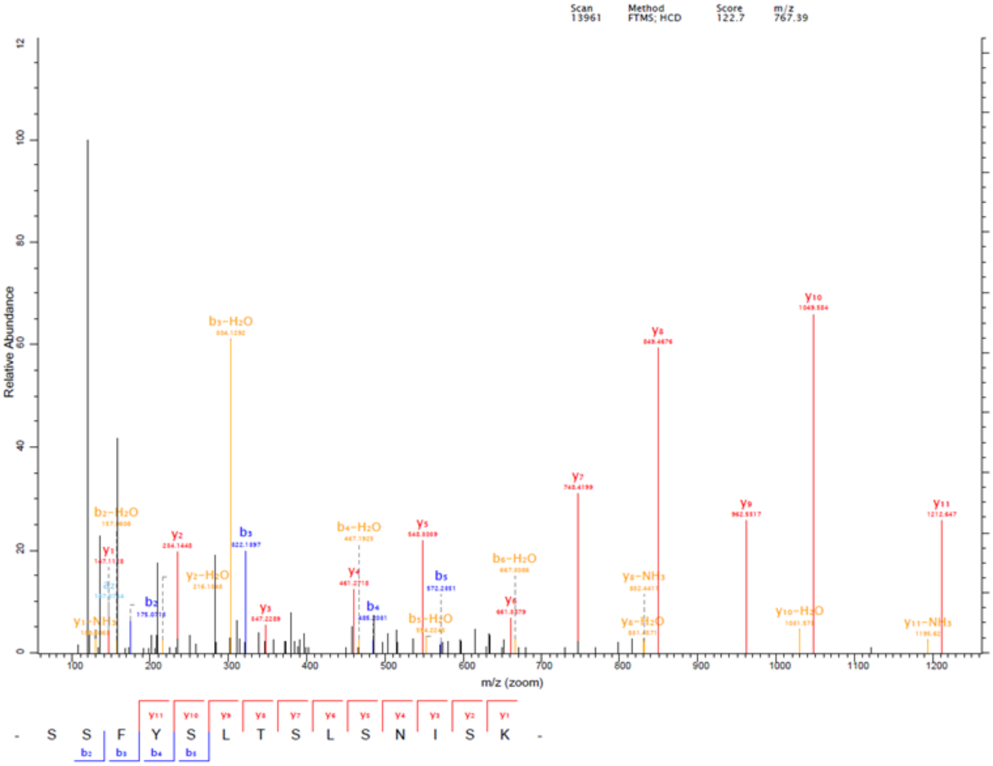

Scan 13961
Method FTMS; HCD
Score 122.7
m/z 767.39
Relative Abundance
m/z (zoom)
b3-H2O
y10
y8
y7
y9
y11
b2-H2O
y1
y2
b3
b4-H2O
y5
y4
b2
y2-H2O
y3
b4
b5
b6-H2O
y6
b5-H2O
y8-NH3
y8-H2O
y10-H2O
y11-NH3
y1-NH3
- S S F Y S L T S L S N I S K -

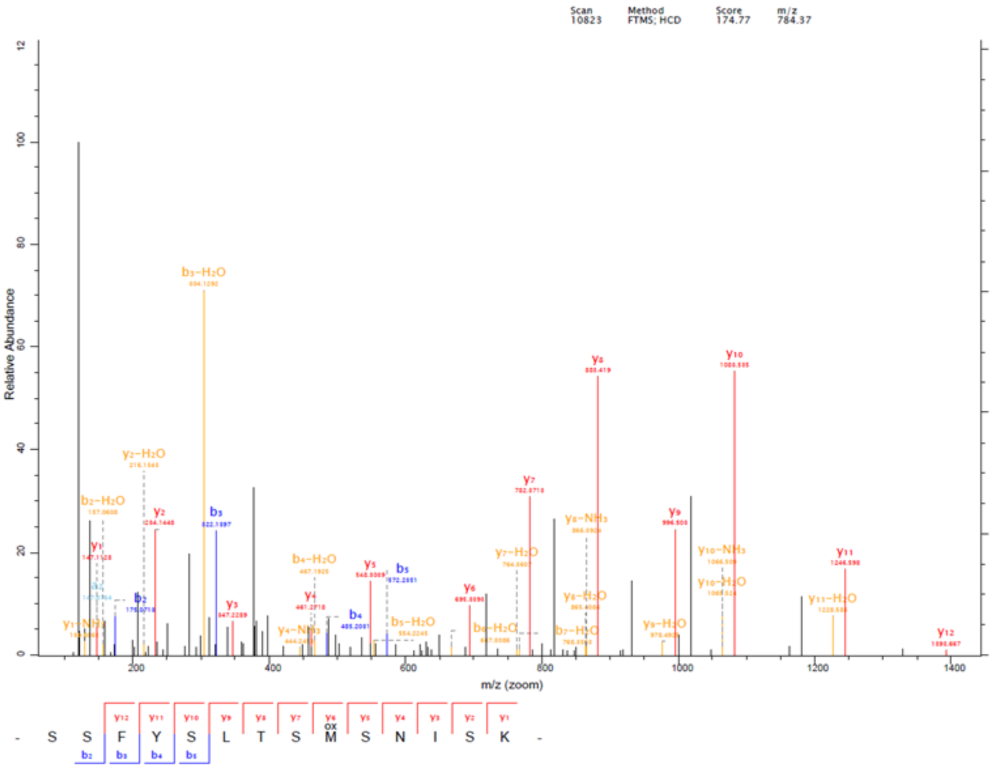

Scan 10823
Method FTMS; HCD
Score 174.77
m/z 784.37
Relative Abundance
m/z (zoom)
- S S F Y S L T S M S N I S K -

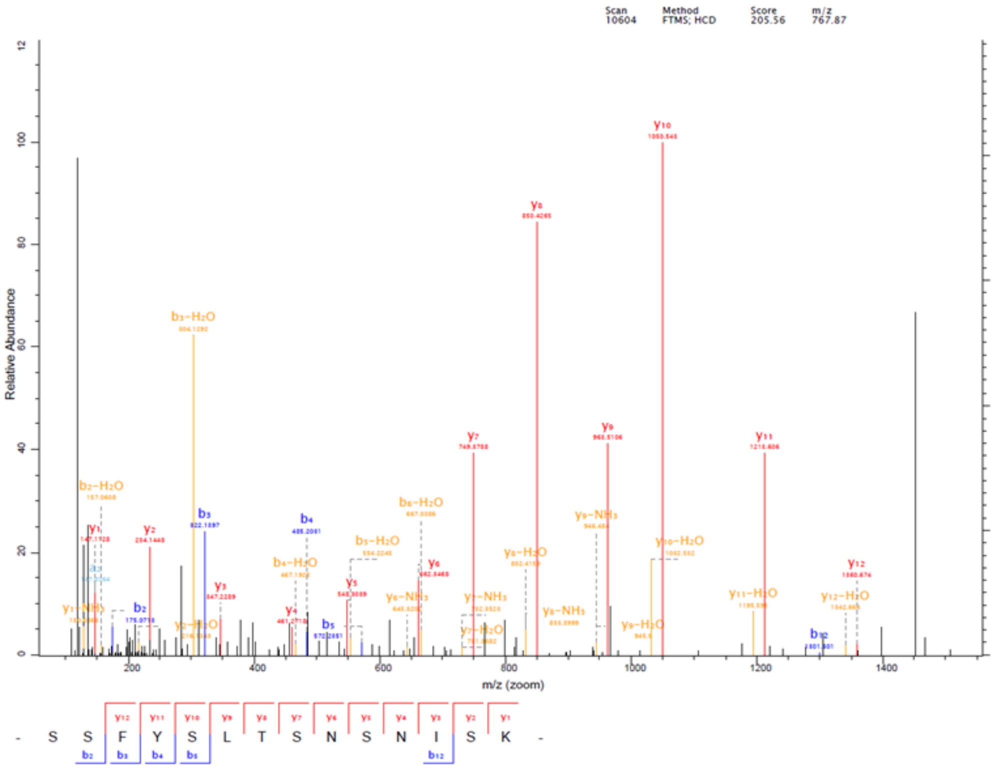

Scan
10604
Method
FTMS; HCD
Score
205.56
m/z
767.87
Relative Abundance
m/z (zoom)
0
20
40
60
80
100
200
400
600
800
1000
1200
1400
y10
y8
b3-H2O
y7
y9
y11
b2-H2O
b3
b4
b6-H2O
y1
y2
b5-H2O
y6
y8-H2O
y9-NH3
y10-H2O
y12
y3
y5
y11-H2O
y12-H2O
b2
y4
b5
y6-NH3
y7-NH3
y8-NH3
y7-H2O
y9-H2O
b12
- S S F Y S L T S N S N I S K -
y12 y11 y10 y9 y8 y7 y6 y5 y4 y3 y2 y1
b2 b3 b4 b5 b12

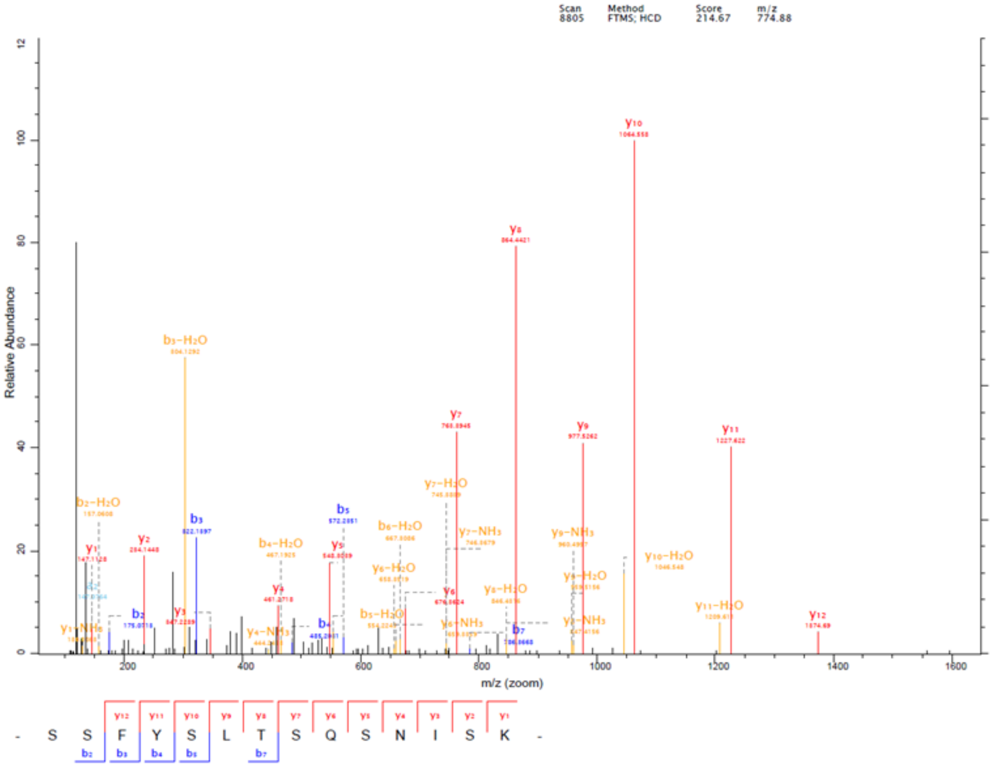
Scan 8805
Method FTMS; HCD
Score 214.67
m/z 774.88
Relative Abundance
m/z (zoom)
- S S F Y S L T S Q S N I S K -

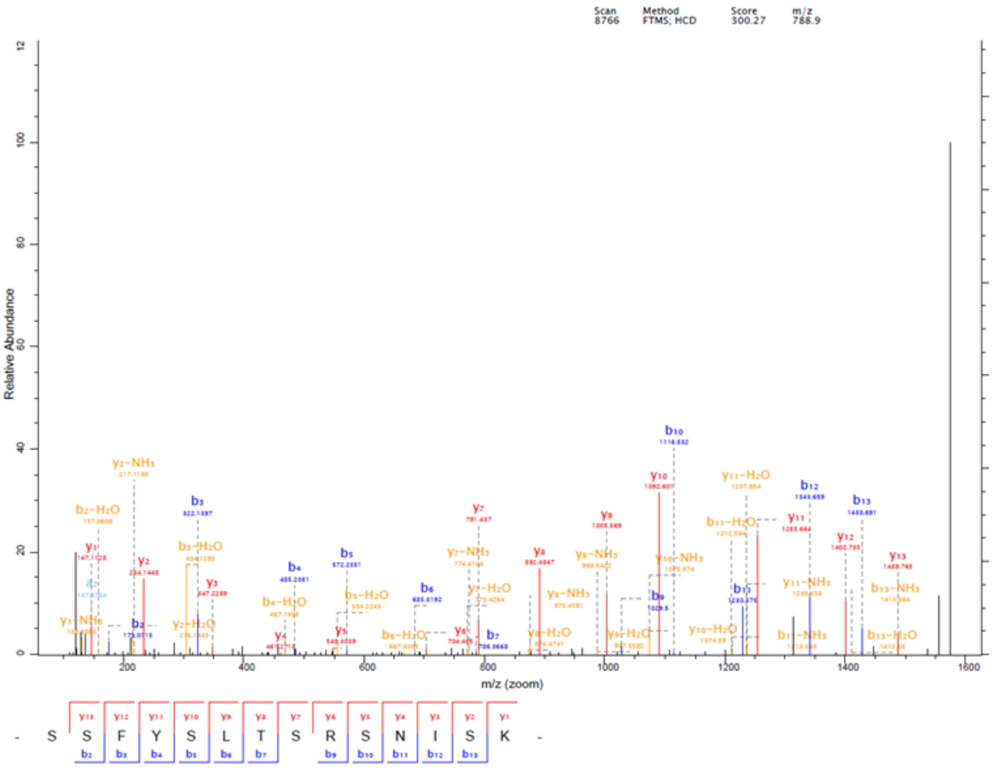
Scan 8766
Method FTMS; HCD
Score 300.27
m/z 788.9
Relative Abundance
m/z (zoom)
- S S F Y S L T S R S N I S K -

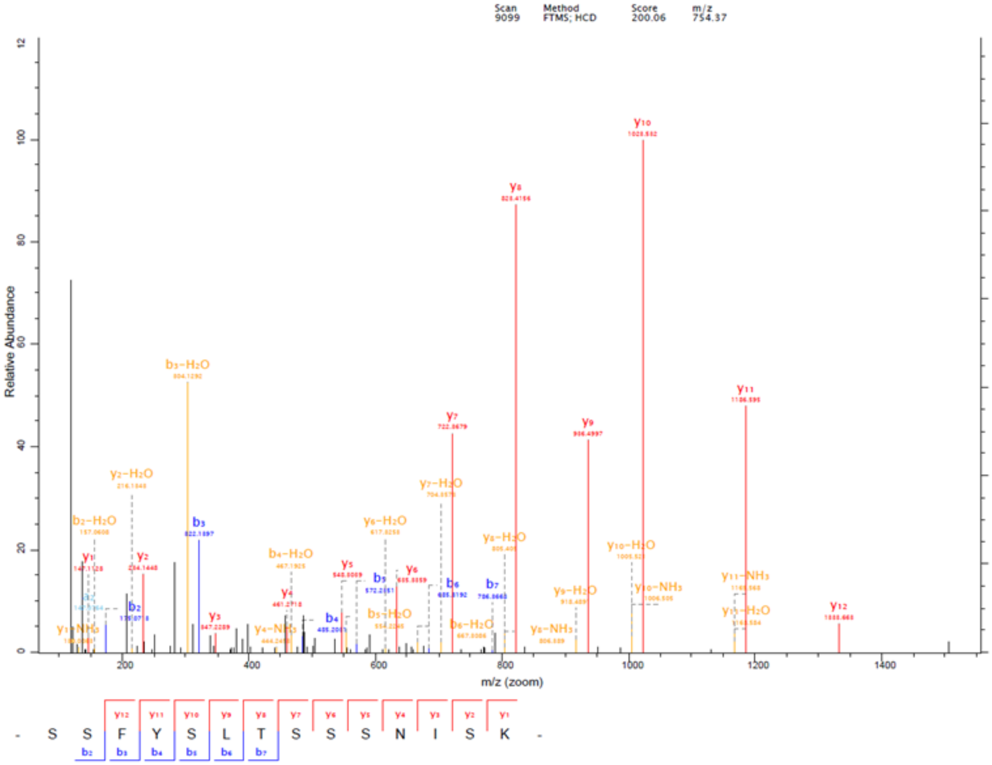
Scan 9099
Method FTMS; HCD
Score 200.06
m/z 754.37
Relative Abundance
m/z (zoom)
- S S F Y S L T S S S N I S K -

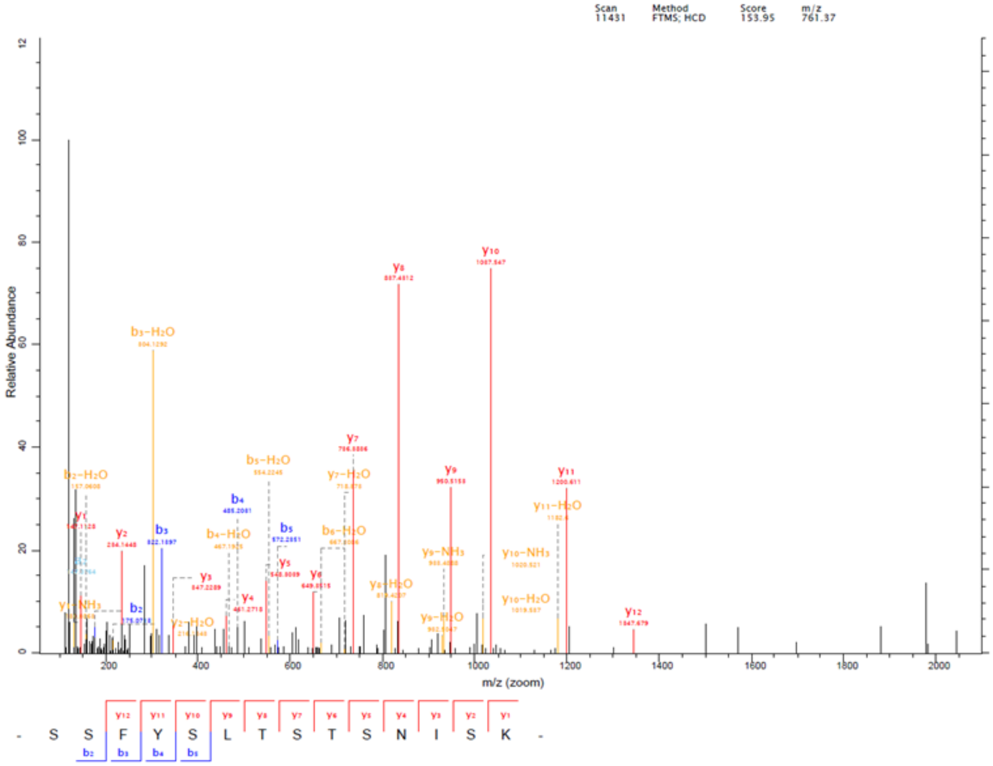
Scan 11431
Method FTMS; HCD
Score 153.95
m/z 761.37
Relative Abundance
m/z (zoom)
y10
y8
b3-H2O
y7
b5-H2O
y9
y11
b2-H2O
y7-H2O
b4
y11-H2O
b3
b5
y2
b4-H2O
b6-H2O
y8-NH3
y10-NH3
y5
y3
y6
y10-H2O
y4
y12
b2
y2-H2O
- S S F Y S L T S T S N I S K -

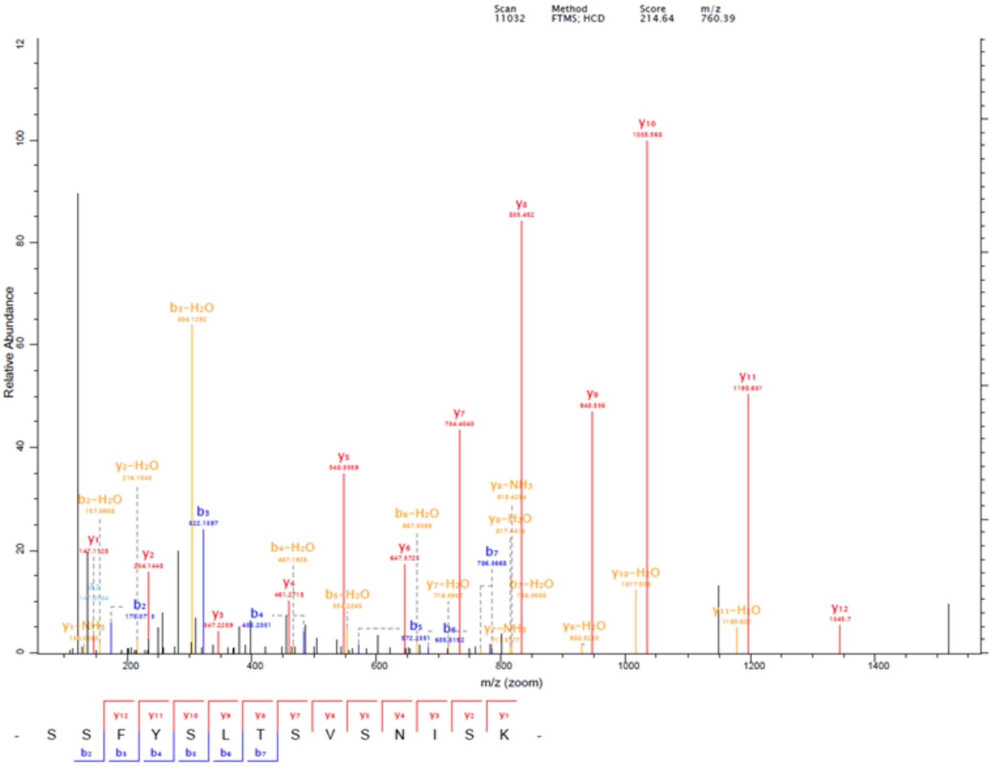

Scan 11032
Method FTMS; HCD
Score 214.64
m/z 760.39
Relative Abundance
m/z (zoom)
y10
y8
y11
b3-H2O
y9
y7
y5
y2-H2O
b2-H2O
b3
b6-H2O
y8-NH3
y6-H2O
y1
y2
b4-H2O
y6
b7
y4
y7-H2O
b7-H2O
y10-H2O
b5-H2O
b2
y11-H2O
y12
y3
b4
y1-NH3
b5
b6
y9-H2O
y7-NH3
S S F Y S L T S V S N I S K

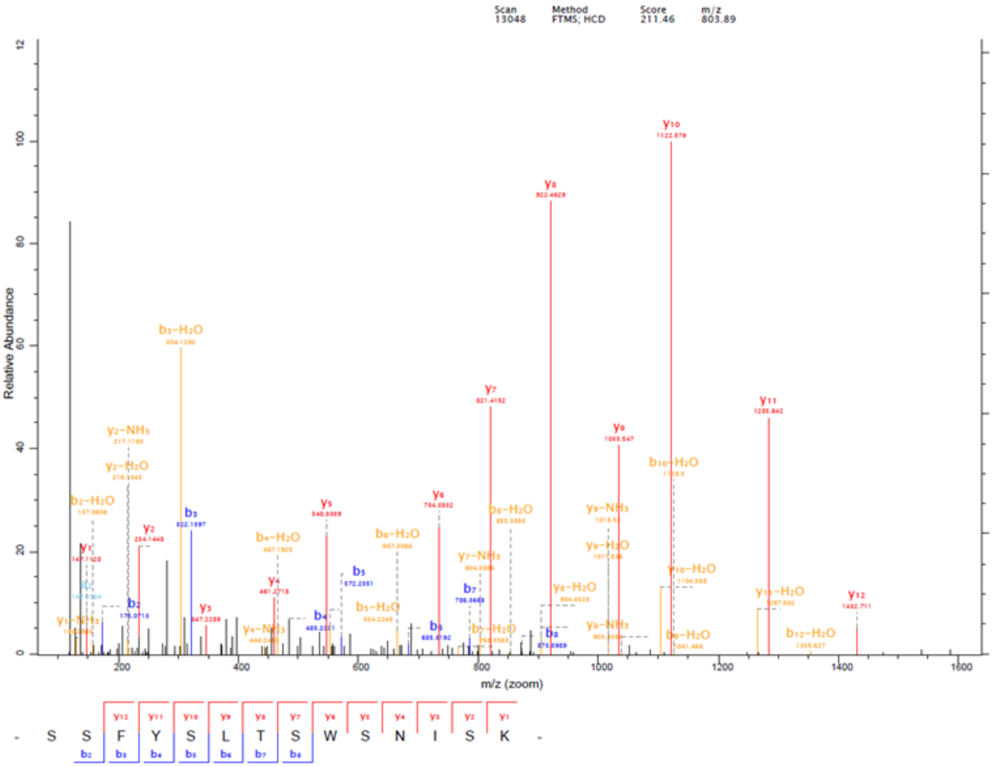
Scan 13048
Method FTMS; HCD
Score 211.46
m/z 803.89
Relative Abundance
m/z (zoom)
y10
y8
b3-H2O
y7
y11
y9
b10-H2O
y2-NH3
y2-H2O
b2-H2O
b3
y5
y6
b8-H2O
y9-NH3
y9-H2O
b4-H2O
b6-H2O
y7-NH3
b5
b7
y8-H2O
y10-H2O
y11-H2O
y12
b12-H2O
b9-H2O
y1
y2
b2
y3
y4
b4
b5-H2O
b6
b8
S S F Y S L T S W S N I S K

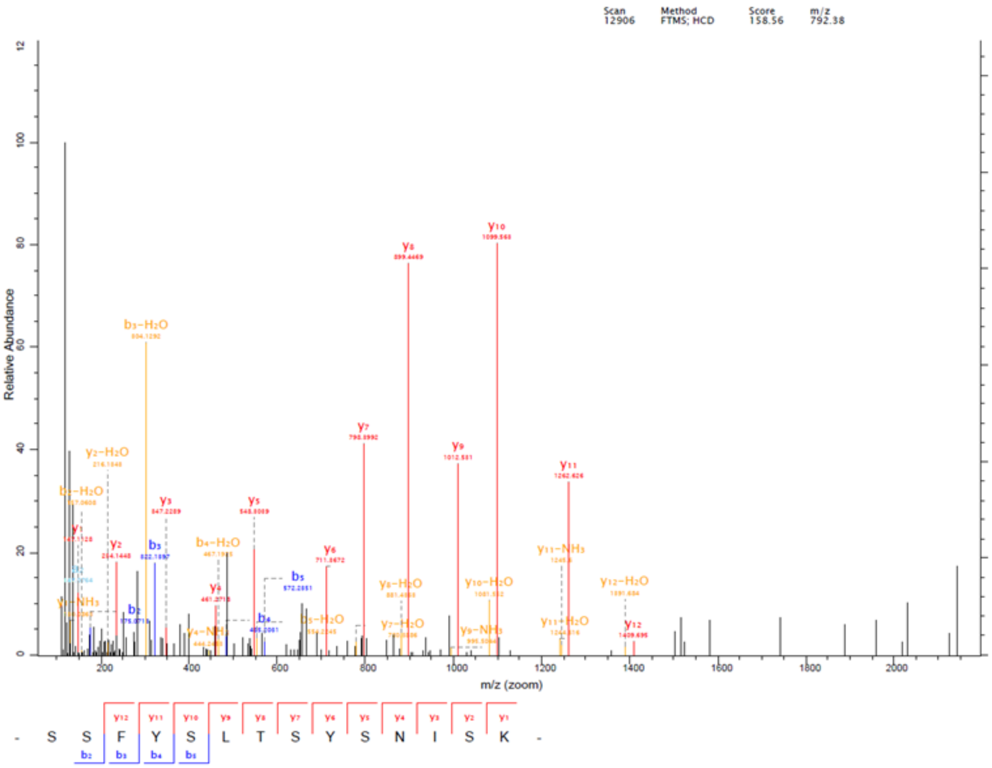
Scan
12906
Method
FTMS; HCD
Score
158.56
m/z
792.38
Relative Abundance
m/z (zoom)
y10
y8
b3-H2O
y7
y9
y11
y2-H2O
y3
y5
b4-H2O
y2
b3
y6
y11-NH3
y10-H2O
y12-H2O
b5
y4
b2
b4
y12
- S S F Y S L T S Y S N I S K -
